# Developmental origin of oligodendrocytes determines their function in the adult brain

**DOI:** 10.1038/s41593-024-01666-8

**Published:** 2024-06-07

**Authors:** Sarah Foerster, Elisa M. Floriddia, David van Bruggen, Petra Kukanja, Bastien Hervé, Shangli Cheng, Eosu Kim, Benjamin U. Phillips, Christopher J. Heath, Richa B. Tripathi, Cody Call, Theresa Bartels, Katherine Ridley, Björn Neumann, Laura López-Cruz, Abbe H. Crawford, Cian J. Lynch, Manuel Serrano, Lisa Saksida, David H. Rowitch, Wiebke Möbius, Klaus-Armin Nave, Matthew N. Rasband, Dwight E. Bergles, Nicoletta Kessaris, William D. Richardson, Timothy J. Bussey, Chao Zhao, Gonçalo Castelo-Branco, Robin J. M. Franklin

**Affiliations:** 1 Wellcome-MRC Cambridge Stem Cell Institute, University of Cambridge, Cambridge, UK; 2 Altos Labs, Cambridge Institute of Science, Cambridge, UK; 3 Laboratory of Molecular Neurobiology, Karolinska Institutet, Stockholm, Sweden; 4 Ming Wai Lau Centre for Reparative Medicine, Stockholm and Hong Kong nodes, Karolinska Institutet, Stockholm, Sweden; 5 Behavioral and Clinical Neuroscience Institute, University of Cambridge, Cambridge, UK; 6 Department of Psychiatry, Institute of Behavioral Science in Medicine, Yonsei University College of Medicine, Seoul, South Korea; 7 Department of Physiology, Development and Neuroscience, University of Cambridge, Cambridge, UK; 8 School of Life, Health and Chemical Sciences, The Open University, Milton Keynes, UK; 9 Wolfson Institute for Biomedical Research, University College London, London, UK; 10 Department of Neuroscience, Johns Hopkins University School of Medicine, Baltimore, MD USA; 11 Department of Paediatrics, University of Cambridge, Cambridge, UK; 12 Department of Physiology and Pharmacology and Robarts Research Institute, Schulich School of Medicine & Dentistry, University of Western Ontario, London, ON Canada; 13 Department of Neurogenetics, Max Planck Institute for Multidisciplinary Sciences, Göttingen, Germany; 14 Department of Neuroscience, Baylor College of Medicine, Houston, TX USA

**Keywords:** Stem cells, Differentiation

## Abstract

In the mouse embryonic forebrain, developmentally distinct oligodendrocyte progenitor cell populations and their progeny, oligodendrocytes, emerge from three distinct regions in a spatiotemporal gradient from ventral to dorsal. However, the functional importance of this oligodendrocyte developmental heterogeneity is unknown. Using a genetic strategy to ablate dorsally derived oligodendrocyte lineage cells (OLCs), we show here that the areas in which dorsally derived OLCs normally reside in the adult central nervous system become populated and myelinated by OLCs of ventral origin. These ectopic oligodendrocytes (eOLs) have a distinctive gene expression profile as well as subtle myelination abnormalities. The failure of eOLs to fully assume the role of the original dorsally derived cells results in locomotor and cognitive deficits in the adult animal. This study reveals the importance of developmental heterogeneity within the oligodendrocyte lineage and its importance for homeostatic brain function.

## Abstract (web summary)

Here the authors show that ventrally derived oligodendrocytes (OLs) can myelinate areas usually populated by dorsally derived OLs but cannot functionally compensate, as animals populated only by ventrally derived OLs show locomotor and cognitive deficits.

## Main

Oligodendrocytes (OLs) are the myelin sheath-forming cells of the central nervous system responsible for saltatory action potential propagation^1–3^, neurotransmitter secretion^4^ and providing lactate to axons for aerobic ATP production^5,6^. In recent years, OLs have emerged as central players in the functional plasticity of the adult central nervous system, contributing, for example, to the acquisition of new complex motor skills^7,8^, the improvement of memory function^9–11^ and behavior after social isolation^12^. OLs have different developmental origins^13^. In the mouse embryonic forebrain, developmentally distinct oligodendrocyte progenitor cell (OPC) populations emerge from three distinct regions in a spatiotemporal gradient from ventral to dorsal. An early population of OPCs arises from the ventral medial ganglionic eminence and anterior entopeduncular (MGE-AEP) area at embryonic day (E) 12.5, whereas, at E15.5, a second population of OPCs arises from the lateral and caudal ganglionic eminences (LGE-CGEs)^13^. Subsequently, a third OPC population, generated dorsally from cortical progenitors, populates the entire cerebral cortex but not the ventral telencephalon^13^. The earliest-formed OPCs exhibit a unidirectional contact repulsion on interneurons, thereby guiding interneurons away from the vasculature and allowing their migration to the cortical layers^14^. However, the number of first-wave OPCs is substantially decreased during postnatal development^13^, with the result that the adult brain is populated by OPCs mostly from the second and third waves. Ventrally derived and dorsally derived OPCs have indistinguishable electrical properties^15,16^. However, the origin of OPCs determines their regenerative potential^17^. Although it is well established that specific functional roles of neurons and astrocytes are closely related to their developmental origins^18–22^, it is unclear whether OL ontogeny also relates to function.

## Results

### Loss of dorsal OLCs is compensated by ectopic ventral OLCs

**Fig. 1:**
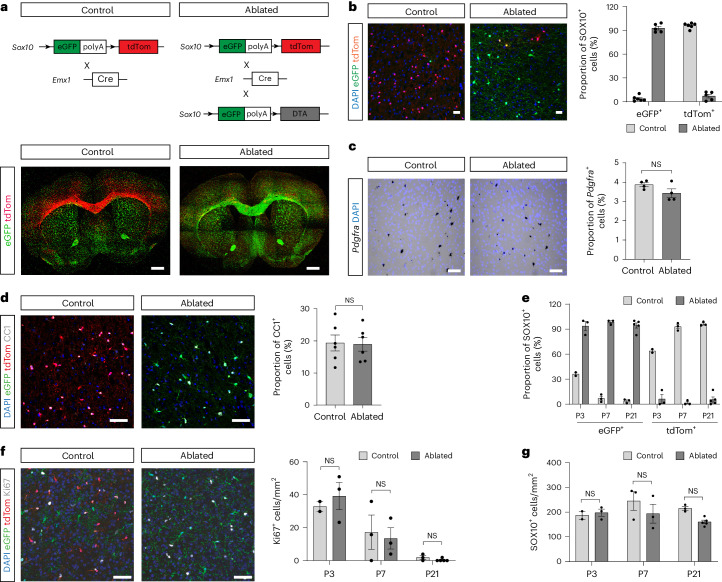
Genetic ablation of dOPCs can be fully restored by cells from ventral origin. **a**, Schematic strategy for genetic fate mapping and ablation of dOLCs. Low-power images showing distribution of dorsally derived tdTom^+^ and ventrally derived eGFP^+^ cells in control (left) and ablated (right) adult mouse forebrain (images were tiled). **b**, Images and quantification of SOX10^+^ OLCs of both dorsal and ventral origin in cerebral cortex from control and ablated adult animals showing the extent of ablation of dOLCs and replacement by vOLCs. P90: control: *n* = 6; ablated: *n* = 5. **c**,**d**, OLC ablation does not alter numbers of *Pdgfra*^*+*^ OPCs (**c**) and mature CC1^+^ OLs (**d**) in adult cortex (all layers). P90: *n* = 4, *P* = 0.904 (**c**); *n* = 6, *P* = 0.126 (**d**), unpaired *t*-test (two-sided). **e**, Relative proportions of SOX10^+^ OLCs that are tdTom^+^ or eGFP^+^ in control and ablated animals. P3, P7 and P21: P3: control: *n* = 2, ablated: *n* = 3; P7: *n* = 3; P21: control: *n* = 3, ablated: *n* = 5. **f**, Images and quantification of Ki67^+^ proliferating cells in cerebral cortex. P3, P7 and P21: *n* = 3, except P3 control: *n* = 2 and P21 ablated: *n* = 5, *P* > 0.05, two-way ANOVA. **g**, Ablation does not result in changes to the overall densities of SOX10^+^ OLCs during developmental myelination. P3, P7 and P21: *n* = 3, except P3 control: *n* = 2, *P* > 0.05, two-way ANOVA. All data are shown as mean ± s.e.m. Scale bars, 50 μm. NS, not significant.

To address whether this developmental heterogeneity in both time and space has functional implications for OLs, we used dual reporter mice (*Sox10-eGFP-tdTom*)^15^ in which OPCs and their OL progeny are labeled according to their developmental origin. To trace OPCs derived from the dorsal forebrain, we used an *Emx1-Cre* mouse line to activate tdTom, whereas OPCs from the ventral forebrain express eGFP^13^. At postnatal day (P) 3 in the cortex of control mice, approximately 70% of OPCs are dorsally derived (dOPCs), which reaches more than 90% at P7 and remains at this level throughout adulthood (Fig. 1a,b,e).

**Extended Data Fig. 1:**
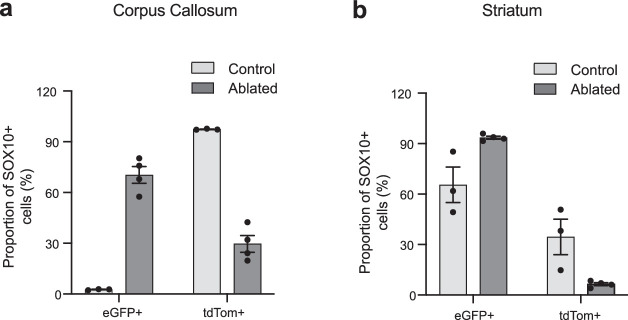
Efficient ablation of dorsal oligodendrocyte lineage cells in other regions of the forebrain. Bar graphs show percentage of oligodendrocyte lineage cells (SOX10 + ) of dorsal (tdTom) or ventral (eGFP) origin in the control and ablated mice in corpus callosum (**a**) or striatum (**b**). P90; control: n = 3, ablated: n = 4. Data are represented as mean ± S.E.M.

dOPCs were specifically ablated by crossing *Emx1-Cre*/*Sox10-eGFP-tdTom* double transgenic mice with a transgenic line (*Sox10-DTA*) expressing diphtheria toxin fragment A (DTA) in OLCs (Fig. 1a). In adult animals, we observed an ablation of 70–85% of dorsally derived oligodendrocyte lineage cells (dOLCs) in the cerebral cortex (Fig. 1b,e), corpus callosum (CC) (Extended Data Fig. 1a) and striatum (Extended Data Fig. 1b). We undertook further analysis in the cerebral cortex. Whereas the tdTom^+^ dOLCs declined within the cerebral cortex in ablated mice compared to non-ablated controls, there was a relative increase in the number of eGFP^+^ cells of ventral origin: 90% of OLCs were eGFP^+^ at P3, a percentage that remained stable at P7 and P21 (Fig. 1b,e). We termed these eGFP^+^ cells that are abundant in areas normally predominantly occupied by tdTom^+^ dorsally derived cells as ectopic oligodendrocyte lineage cells (eOLCs). Despite the substantial shift in the proportion of eGFP^+^ and tdTom^+^ cells toward eGFP^+^ cells of ventral origin, we found no change in the overall number of *Pdgfra*^+^ OPCs (Fig. 1c) and CC1^+^ OLs (Fig. 1d) in the cerebral cortex in adult animals. The ablation of dOLCs was not accompanied by a change in proliferative response in eOPCs (Fig. 1f), which, together with the maintenance of a normal OL density (Fig. 1g), suggests that ventral OL cells have already populated the developing cortex in sufficient numbers before birth, a conclusion supported by data reported by Kessaris et al.^13^. Thus, numbers of OLCs are sustained after dOLC ablation by the persistence of OLCs of ventral origin.

### eOLs in dorsal forebrain exhibit subtle myelination changes

**Fig. 2:**
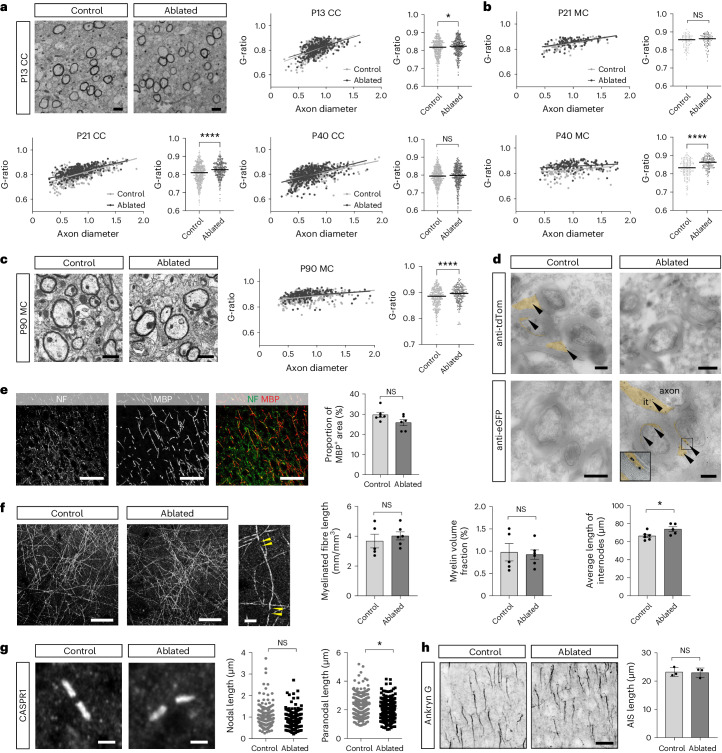
Myelination by eOLC-derived OLs exhibits a minor spatially and temporally restricted reduction in sheath thickness. **a**, Quantitative analysis of G-ratio of myelinated axons in the CC of control and ablated mice. Electron microscopy images are of similar areas of CC at P13. P13, P21 and P40, *n* = biological replicates, *N* = number of axons. P13: control: *n* = 5, *N* = 498, ablated: *n* = 3, *N* = 330, *P* = 0.0419; P21: control: *n* = 4, *N* = 560, ablated, *n* = 3, *N* = 342, *P* < 0.0001; P40: control: *n* = 4, *N* = 553, ablated: *n* = 3, *N* = 421, *P* = 0.1315. Mann–Whitney test. **b**, G-ratios of axons in motor cortex (MC) layers 5–6. P21 and P40. P21: control: *n* = 3, *N* = 91, ablated: *n* = 3, *N* = 92, *P* = 0.195; P40: control: *n* = 4, *N* = 152, ablated: *n* = 3, *N* = 139, *P* < 0.0001. Mann–Whitney test. **c**, G-ratios in MC layers 5–6 in mice. P90: control: *n* = 4, *N* = 385, ablated: *n* = 3, *N* = 195, *P* < 0.0001. Mann–Whitney test. **d**, Immunogold electron microscopy on cryosections for eGFP and tdTom in control and ablated mice. Arrowheads, myelinated axon; it, inner tongue; ot, outer tongue; orange, OL. P90: *n* = 1. **e**, MBP and NF staining in the MC layers 5–6 in control and ablated animals. P90: *n* = 6, *P* = 0.0741, unpaired *t*-test (two-sided). **f**, SCoRe microscopy of the cerebral cortex layer 1 in control and ablated mice. Arrowhead = node of Ranvier. P90: for myelin fiber lengths and volume fraction: control: *n* = 6; ablated: *n* = 5, *P* = 0.537 and *P* = 0.823, respectively, for average internode lengths: *n* = biological replicates, *N* = number of internodes; control: *n* = 6, *N* = 294, ablated: *n* = 5, *N* = 248, *P* = 0.037, Mann–Whitney test. **g**, Super-resolution microscopy images of CASPR1^+^ paranodes of myelinated axons in the cortex in control and ablated animals. P90: *n* = biological replicates, *N* = number of nodes and paranodes; nodes: *n* = 3, control: *N* = 208, ablated: *N* = 171, *P* = 0.139, unpaired *t*-test (two-sided); paranodes: *n* = 3, control: *N* = 463, ablated: *N* = 406, *P* < 0.0001, unpaired *t*-test (two-sided). **h**, Quantification of Ankyrin G^+^ axon initial segments (AIS) length in the pyramidal neurons in the MC in control and ablated mice. P90: *n* = biological replicates, *N* = number of AIS, control: *n* = 3, *N* = 130, ablated: *n* = 3, *N* = 137, *P* = 0.624, Mann–Whitney test. The graphs in **e**–**h** show mean ± s.e.m. and are overlayed with individual data points. *P < 0.05; ****P < 0.001. Scale bars, 2 μm in **a** and **c**; 50 μm in **e**; 1 μm in **d**; 2 μm in **g**; and 20 μm in **f** and **h**. NS, not significant.

We next assessed myelination in the CC and cerebral cortex in control and ablated animals. In the CC, we found a subtle and transient increase in the G-ratio (the ratio between the inner axon radius and the outer myelinated axon radius) in the ablated animals at P13 and P21, but not at P40, indicating that eOLs formed slightly thinner myelin sheaths (Fig. 2a). In contrast, in deep cortical layers of ablated animals, although there was no difference in G-ratios at P21, there were higher G-ratios associated with smaller-diameter axons at P40 and P90 (Fig. 2b,c). Using immuno-electron microscopy to detect the fluorescent marker proteins tdTom and eGFP, we found no difference in the compaction of myelin sheaths made by cells of either dorsal or ventral origin in adult animals (Fig. 2d). The overall level of myelination assessed by MBP immunohistochemistry staining in neocortical layers 5–6 also remained unchanged at P90 (Fig. 2e). Spectral confocal reflectance (SCoRe) imaging of cortical layer 1 showed no difference in the measured length of myelinated axons present within the sample fields or total amount of myelin (myelin volume fraction) within a unit area between control and ablated animals (Fig. 2f). However, we did observe an increase in average internodal length in adult ablated animals (Fig. 2f). Using super-resolution microscopy, we found no difference in nodal length between control and ablated animals. Nevertheless, a decrease in paranodal length was detected in adult ablated animals (Fig. 2g), an expected finding given the observation that a proportion of axons in these animals exhibit thinner myelin sheaths^23^. Immunostaining for Ankyrin G showed that axon initial segments of neocortical pyramidal neurons were unaltered between control and ablated adult animals (Fig. 2h). Thus, ventrally derived OPCs (vOPCs) did not entirely compensate for the lack of dOPCs, forming an ectopic population of neocortical eOLs with subtle myelination differences from the myelination pattern mediated by OLs of dorsal origin (Figs. 1 and 2).

### eOL myelination of the dorsal forebrain leads to motor changes

**Fig. 3:**
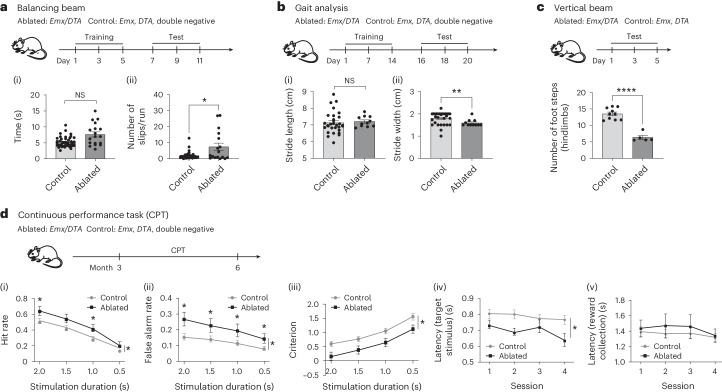
Ablation of dOPCs causes behavioral deficits. **a**, Balancing beam test: (i) beam traverse time: control: *n* = 42, ablated: *n* = 18, *P* = < 0.01, Mann–Whitney test; (ii) number of hindlimb slips per traverse: control: *n* = 40, ablated: *n* = 17, *P* = 0.05, Kolmogorov–Smirnov test. **b**, Gait analysis: control: *n* = 30, ablated: *n* = 11. (i) Average hind stride length: *P* = 0.71, unpaired *t*-test (two-sided); (ii) average hind stride width: *P* = 0.01, Mann–Whitney test. **c**, Vertical beam test: number of hindlimb steps: control: *n* = 10, ablated: *n* = 5, *P* = < 0.0001, Mann–Whitney test. **d**, rCPT: simulation duration is the time each stimulus (target or non-target) was shown to each mouse. (i) HR: mixed-effect model: main effect of genotype, *P* = 0.037; main effect of duration, *P* < 0.0001; genotype by duration interaction, *P* = 0.005; simple main effects of genotype at 2.0 s, 1.0 s and 0.5 s, *P* < 0.05. (ii) FAR: mixed-effect model: main effect of genotype, *P* = 0.023; main effect of duration, *P* < 0.0001; genotype by duration interaction, *P* = 0.003; simple main effects of genotype at each duration condition, *P* < 0.05. (iii) Criterion: mixed-effect model: main effect of genotype, *P* = 0.025; main effect of duration, *P* < 0.0001; genotype by duration interaction, *P* = 0.775; simple main effects of genotype at each duration condition, *P* < 0.05. (i–iii) Control: *n* = 27, ablated: *n* = 11. (iv) Response latency to ‘target’ stimulus: mixed-effect model: main effect of genotype, *P* = 0.033; main effect of session, *P* = 0.017; genotype by session interaction, *P* = 0.158. (v) Reward collection latency: mixed-effect model: main effect of genotype, *P* = 0.554; main effect of session, *P* = 0.340; genotype by session interaction, *P* = 0.862. (iv,v) Control: *n* = 27, ablated: *n* = 11. All data are mean ± s.e.m. Two cohorts of animals were used for the locomotor and cognitive testing, respectively. All locomotor tests were performed consecutively. Training of both cohorts started at P90. NS, not significant.

**Extended Data Fig. 2:**
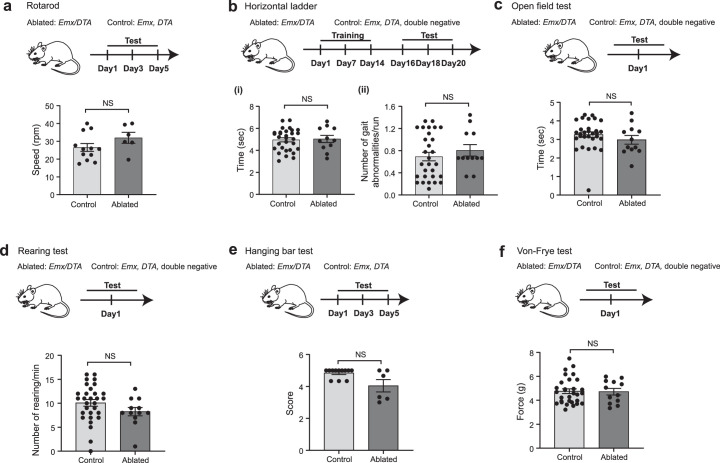
Ablated mice do not show deficits in any other locomotor task and show no signs of anxiety, aberrant muscle tonus or sensation deficits. (**a**) Accelerating rotarod: Rod rotation speed at which mice fell off the rod. N = 3; n = 12 (control) and n = 6 (ablated) mice/condition, p-value = <0.17 unpaired t-test (two-sided). (**b**) Horizontal ladder: (i) Time mice needed to cross the ladder. N = 3; control: n = 30, ablated: n = 11 mice/condition, p-value = <0.01, Mann-Whitney test; (ii) Number of gait abnormalities (including misses, slip offs) per run. N = 3; control: n = 29, ablated: n = 12 mice/condition, p-value = 0.83, unpaired t-test (two-sided). (**c**) Open field test: Time a mouse spent at the wall of the test chamber. N = 1; control: n = 30, ablated: n = 12 mice/condition, p-value = 0.22, Mann-Whitney test. (**d**) Rearing test: Number of rearings per test. N = 1; control: n = 30, ablated: n = 12 mice/condition, p-value = 0.15, unpaired t-test (two-sided). (**e**) Hanging bar test: Score indicates the number of seconds each mouse holds on to the bar. A higher score indicates a better performance. N = 3; control: n = 12, ablated: n = 6 mice/condition, p-value = 0.06, Mann-Whitney test. (**f**) Von-Frey test: Force needed to be applied to hind paw before paw retraction. N = 5; control: n = 30, ablated: n = 12 mice/condition, p-value = 0.92, unpaired t-test (two-sided). All locomotor tests were performed consecutively. Training of the mice was started at P90. Data are represented as mean ± S.E.M.

**Extended Data Fig. 3:**
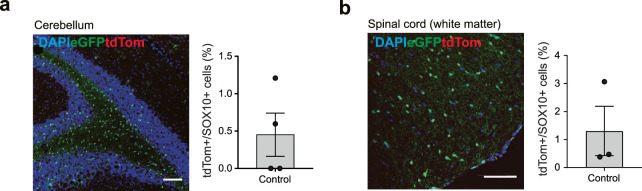
Very few dorsally-derived oligodendrocyte lineage cells are detected in cerebellum and spinal cord. Distribution and quantification of SOX10+ oligodendrocyte lineage cells of dorsal (tdTom) or ventral (eGFP) origin, in cerebellum (**a**) and spinal cord white matter (**b**). P90; (a) n = 4, (b) n = 3. Data are represented as mean ± S.E.M. Scale bars represent 100μm.

To investigate whether these subtle myelin changes induce functional abnormalities or if eOLCs can still functionally compensate for dOLCs, we performed a series of locomotor tests because there was substantial replacement of dOLCs with eOLCs within the motor cortex. Mice with cortices populated by eOLCs (ablated mice) showed significant deficits in horizontal balancing beam walking (Fig. 3ai,ii and Supplementary Video 1), reduced stride width (gait test) (Fig. 3bi,ii) and impaired ability to coordinate fast movement (vertical beam test) (Fig. 3c). However, they showed no deficits in the rotarod (Extended Data Fig. 2a) and horizontal ladder (Extended Data Fig. 2bi,ii) tests and had typical vigilance (Extended Data Fig. 2c,d), muscle strength (Extended Data Fig. 2e) and touch sensation (Extended Data Fig. 2f). Because *Emx1*-Cre is specific to the forebrain, with only negligible numbers of SOX10^+^/tdTom^+^ OLCs in the cerebellum and spinal cord (Extended Data Fig. 3), we reasoned that the locomotor phenotype was due to cerebral cortical dysfunction.

### eOL myelination of dorsal forebrain leads to cognitive changes

**Extended Data Fig. 4:**
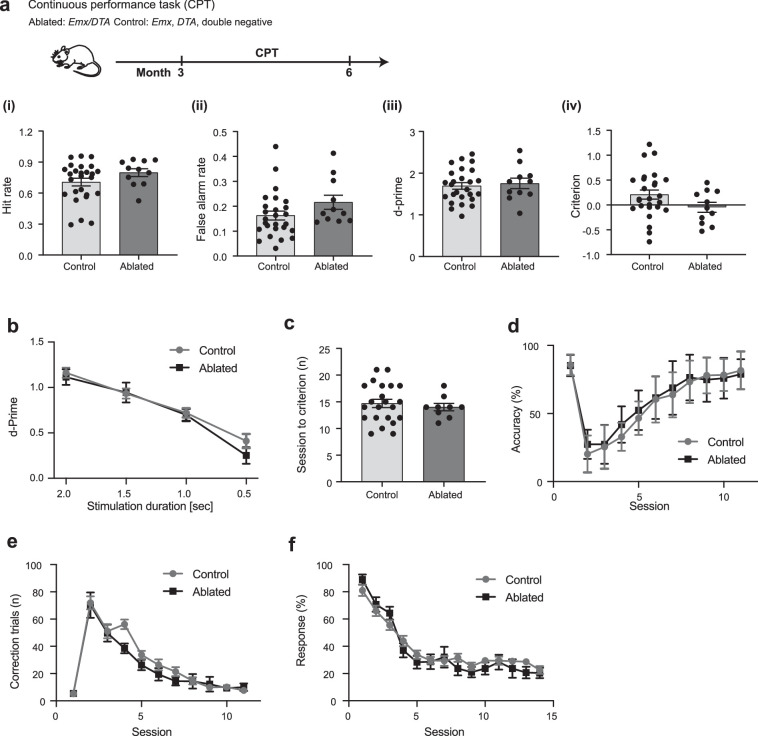
Continuous performance task (rCPT) did not reveal altered attentive behavior in ablated adult mice at baseline and ablation of dOPCs does not induce deficit in visual discrimination, reversal learning and extinction learning. (**a**) rCPT at baseline (for parameters see methods): N = 1; control: n = 26, ablated: n = 11 mice/condition. (i) Hit rate at baseline. p-value = 0.15, unpaired t-test (two-sided). (ii) False alarm rate at baseline. p-value = 0.08 Mann-Whitney test. (iii) D-Prime at baseline. p-value = 0.68, unpaired t-test (two-sided). (iv) Criterion at baseline. p-value = 0.11, unpaired t-test (two-sided). (**b**) D-Prime with shortened SD (for parameters see methods). Mixed-effect model: main effect of genotype, p = 0.459; main effect of duration, p < 0.0001; genotype by duration interaction, p = 0.551. (**c**) Visual discrimination test: Mice learn to discriminate one ‘target’ from one ‘non-target’ stimulus. Session to criterion describes the number of training sessions needed to successfully discriminate the two visual stimuli. N = 1; control: n = 27, ablated: n = 10 mice/condition; control: n = 5, ablated: n = 1 mice/condition did not reach the criterion. p-value = 0.60, unpaired t-test (two-sided). (d,e) Reversal learning test: Target stimulus becomes non-target stimulus in session 2. (**d**) Percentage of correct responses to new target stimulus. N = 1; control: n = 22, ablated: n = 9 mice/condition. Mixed-effect model: main effect of genotype, p = 0.490; main effect of duration, p < 0.0001; genotype by duration interaction, p = 0.562. (**e**) Number of correction trials due to a response to a non-target stimulus. N = 1; control: n = 26, ablated: n = 11 mice/condition. Mixed-effect model: main effect of genotype, p = 0.186; main effect of duration, p < 0.0001; genotype by duration interaction, p = 0.350. (**f**) Extinction learning test: Response to target stimulus is not rewarded. Percentage of responses to target stimulus per session. N = 1; control: n = 26, ablated: n = 10 mice/condition. Mixed-effect model: main effect of genotype, p = 0.723; main effect of duration, p < 0.0001; genotype by duration interaction, p = 0.121. All data are presented as mean ± S.E.M. Training of the mice was started at P90.

We then tested for cognitive changes in ablated mice using the rodent continuous performance task (rCPT), which assesses attention by requiring mice to discriminate visual ‘target’ and ‘non-target’ stimuli and respond only to the target^24^ (Fig. 3d). Ablated mice showed an increase in both hit rate (HR) (Fig. 3d(i)) and false alarm rate (FAR) (Fig. 3d(ii)), indicating a reduced criterion c (threshold for responding to ‘target’ or ‘non-target’ stimuli) (Fig. 3d(iii)) with normal d′ (perceptual discriminability between the ‘target’ and ‘non-target’ stimuli) (Extended Data Fig. 4b). The observed behavioral outcome (low c and normal d′) likely indicate impulsive responding in ablated mice, a conclusion supported by the observation that changes in criterion c were apparent only when mice were challenged with shortened stimulus durations (SDs). Changes in criterion c were not detected at baseline condition with longer and consistent SD (Extended Data Fig. 4a). The hypothesis that ablated mice show increased impulsivity was further supported by a decrease in response latency (Fig. 3d(iv)) with unaltered reward collection latency (Fig. 3d(v)), ruling out differences in locomotor speed. However, the difference in stimulus response between control and ablated animals could also result from the inability to discriminate shapes or perseveration. Thus, we next conducted tests of visual discrimination (Extended Data Fig. 4c) and reversal learning (Extended Data Fig. 4d,e), which ruled out any deficits in perceptual discrimination and perseveration of ablated mice. Finally, insensitivity to the absence of a reward as a potential cause of the indiscriminate responding was excluded by normal extinction learning (Extended Data Fig. 4f). Collectively, these data suggest that ablated mice exhibit an impulsive phenotype, suggesting impaired prefrontal inhibitory control. This pattern of behavior in the CPT task resembles that after a lesion to the anterior cingulate cortex (ACC) in mice^25^, suggesting that the ACC might be involved in controlling the impulsive phenotype observed in ablated mice.

### Ablation of dOLCs causes minor changes in a neuron subtype

**Fig. 4:**
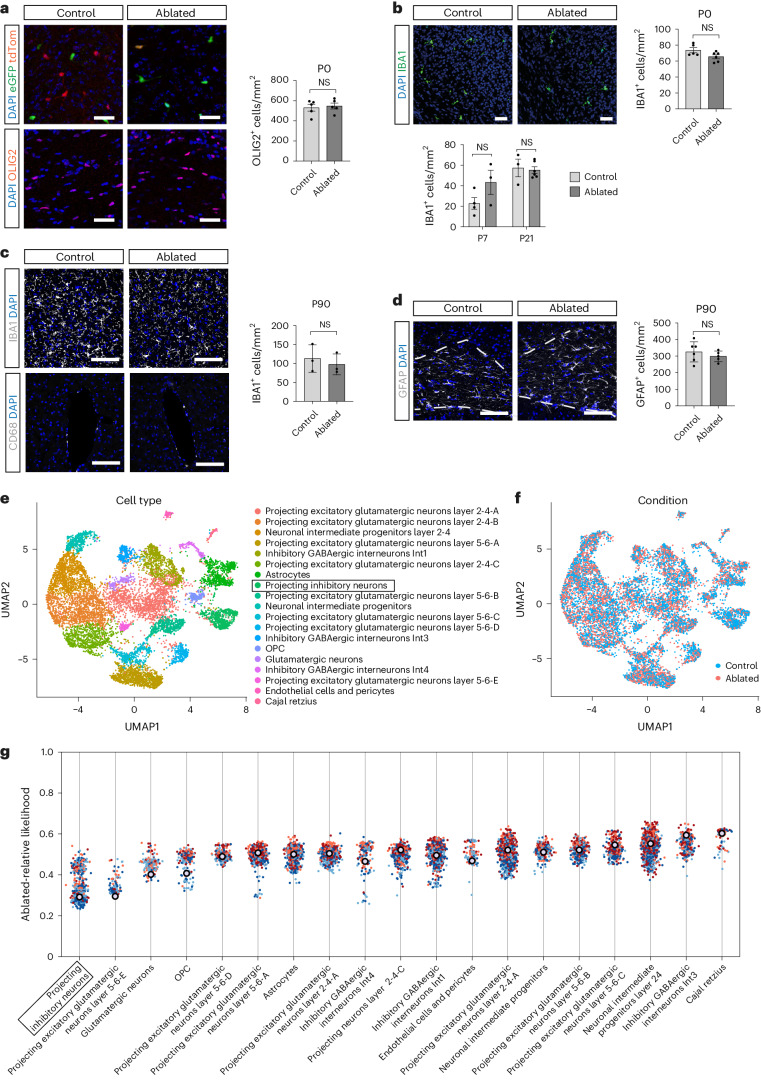
Ablation of dOLCs does not lead to significant alteration in composition of other cell types in the neocortex. **a**, Density of OLIG2^+^ OLCs in CC and cerebral cortex of control and ablated animals. P0: *n* = 5, *P* = 0.735, unpaired *t*-test (two-sided). **b**, Densities of IBA1^+^ microglia in cerebral cortex of control and ablated animals. P0: *n* = 5; P7: control: *n* = 4, ablated: *n* = 3; P21: control: *n* = 3, ablated: *n* = 5, *P* > 0.05, unpaired *t*-test (two-sided). **c**, Densities of IBA1^+^ microglia and CD68^+^ macrophages in cerebral cortex of control and ablated animals. P90: *n* = 3, *P* = 0.5864, unpaired *t*-test (two-sided). **d**, Densities of GFAP^+^ astrocytes in the CC of control and ablated animals. P90: control: *n* = 6, ablated: *n* = 5, *P* = 0.399, unpaired *t*-test. Data are shown as individual values and mean ± s.e.m. and compared by unpaired *t*-test, *P* > 0.05 in all analysis. Scale bars, 50 μm in **a** and **b** and 100 μm in **c** and **d**. **e**–**g**, snRNA-seq of motor cortex tissue dissected from control (*n* = 2) and ablated (*n* = 2) animals at P0 (equivalent to E19) (*N* = 11,052 nuclei). **e**,**f**, UMAPs depicting annotated clusters derived from control and ablated samples. **g**, MELD analysis showed no major transcriptomic changes upon ablation in any of the annotated clusters, apart from moderate differences detected within the population of projecting inhibitory neurons. NS, not significant; UMAP, uniform manifold approximation and projection.

**Extended Data Fig. 5:**
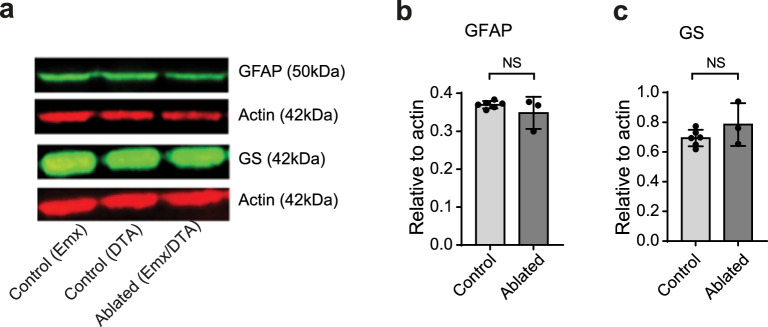
Astrocyte are unchanged in ablated animals. (a-c) Whole-brain western blot analysis of GFAP and glutamine synthetase (GS). P90; control: n = 6, ablated: n = 3, GFAP: p = 0.472, GS: p = 0.388, unpaired t-test (two-sided) with Welch’s test. The full Western Blot image is unavailable.

**Extended Data Fig. 6:**
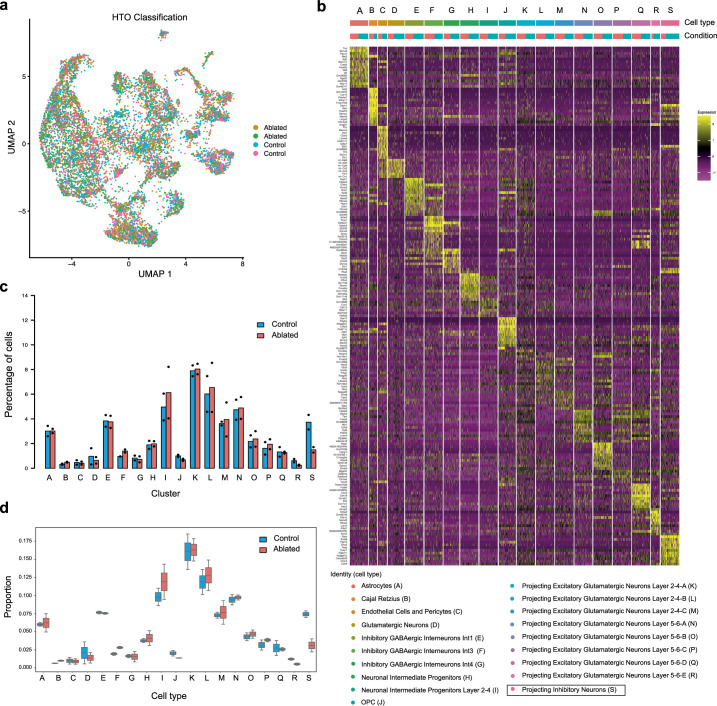
Single nuclear sequencing did not reveal any major bystander effect on brain cells after dOLC ablation. (**a**) Uniform Manifold Approximation and Projection (UMAP) of recovered cells with unique barcoded antibody colored by antibody. Cortical tissue, P0. (**b**) Top 15 Highly Variable Genes (HVGs) for each cell type from Fig. 4e, calculated with Wilcoxon rank sum test, p-value adjusted < 0.01 and log2FC >= 0.25, ranked by log2FC. (**c**) Representative percentage of each cell typecluster in each condition divided by the number of conditions. Bar plots in red and blue correspond to populations recovered from ablated and control samples, respectively. Black dots represent unique antibody-hashed replicates. n = 2. (**d**) scCODA reveals no credible compositional variation from ablated animals.Population of astrocytes was selected as reference for parameters inference. Control condition is indicated in blue, and ablated condition in red. n = 2. In the box plot, the top and bottom whiskers define the maximum and minimum, respectively. The top bound of the box defines the 75ste percentile, the centre line defines the 50ste percentile and the bottom bound of the box defines the 25ste percentile.

**Extended Data Fig. 7:**
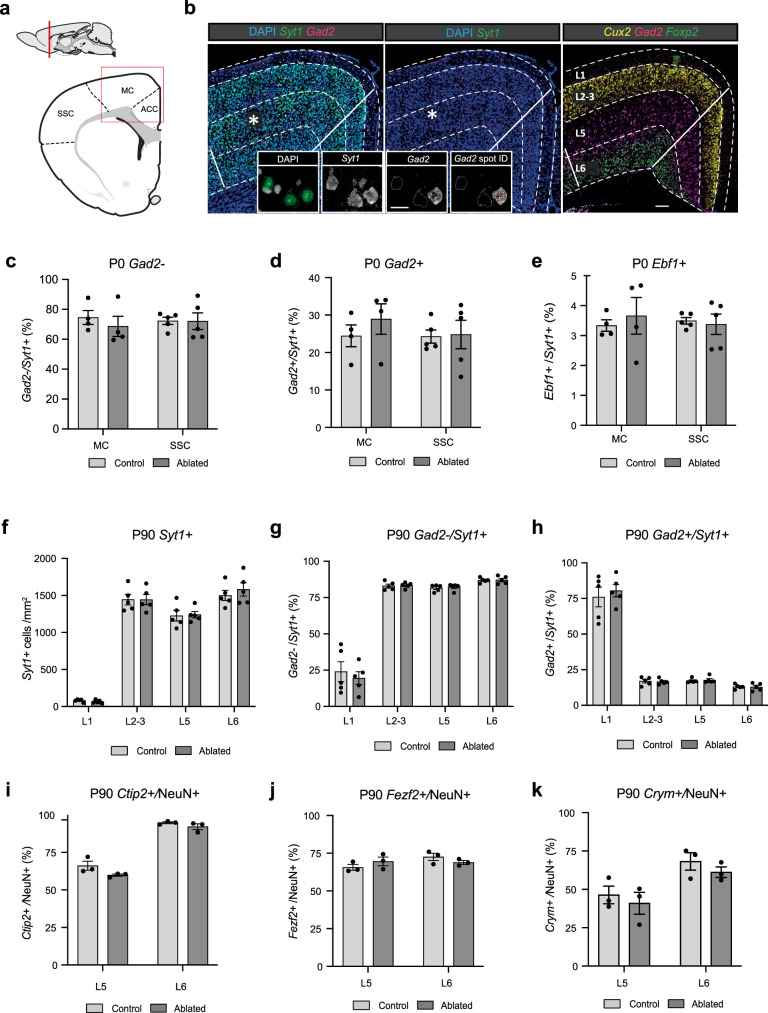
RNAscope analysis did not reveal altered neuronal population in ablated animals. (**a**) Schematic illustration of coronal view of mouse brain for main cortical regions at P90: anterior cingulate cortex (ACC), motor cortex (MC) and somatosensory cortex (SSC). The red line in the sagittal view indicates the level of the sections analyzed. (**b**) Low magnification images of coronal section of MC and ACC regions (red box in (**a**)) co-stained for the pan-neuronal marker Syt1, inhibitory neuron marker Gad2 and neuronal layer markers Cux2, Ctip2 and Foxp2 with RNAscope smFISH. The nuclei are counterstained with DAPI. Scale bar: 200 µm, inset: 20 µm. (c-e) Quantification of proportion of Gad2- excitatory (**c**), Gad2+ inhibitory (**d**) and Ebf1+ projection inhibitory neurons (**e**) co-expressing neuron-specific Syt1 in MC and SSC. No differences were detected between control and dorsally ablated animals. P0; MC: n = 4, SSC: n = 5, p > 0.05, two-way ANOVA. (f-h) In adult brain, there is no difference in the density of Syt1+ (**f**) or the proportion of Gad2- excitatory (**g**) and Gad2+ inhibitory (**h**) neurons between control and ablated animals across the layers of the cerebral cortex, which is shown for motor cortex. P90; control: n = 5, ablated: n = 5, p > 0.05, two-way ANOVA. (i-k) The proportion of Ctip2+ (**i**), Fezf2+ (**j**) and Crym+ (**k**) corticospinal motor neurons co-expressing NeuN in layers 5 and 6 of the adult motor cortex were also unchanged between control and ablated animals. P90; n = 3, p > 0.05, two-way ANOVA. Data are shown as mean +/- S.E.M.

To assess whether the functional deficits that we observed were due to ‘off-target’ effects on cells other than within the OL lineage that had been induced during the ablation process, we performed immunohistochemistry for OLCs, microglial and astrocyte markers (Fig. 4a–d) as well as western blot analysis for astrocyte markers (Extended Data Fig. 5). We did not detect changes in the number of OLCs, microglia or astrocytes at P0 and P90 (Fig. 4a–d and Extended Data Fig. 5). To further investigate the transcriptional landscapes of glial and neuronal populations upon ablation, we performed single-nucleus RNA sequencing (snRNA-seq) with barcoded antibodies^26^ on nuclei isolated from motor cortices at P0, capturing the developmental period when ablation of dOPCs is in progress. Nuclei from distinct samples and biological replicates were uniquely labeled with barcoded antibodies before sequencing to minimize potential batch effects and their impact on sample composition (Fig. 4e,f and Extended Data Fig. 6a,b). Despite the ongoing depletion of dOLCs, we did not detect any differences in proportions of most of the populations at this time point, as assessed by frequency analysis supported with scCODA^27^ (Extended Data Fig. 6c,d), nor in their patterns of gene expression, as assessed by perturbation analysis with MELD^28^ (Fig. 4g). We did not observe microglia in our dataset. However, we found a minor decrease in the proportion of *Ebf1*^+^ projection inhibitory interneurons (cluster Sl; Extended Data Fig. 6c,d). The difference in the number of *Ebf1*^+^ projection inhibitory interneurons was not replicated using RNAscope. RNAscope-based in situ hybridization analysis (Extended Data Fig. 7) indicated no difference in the overall number of excitatory and inhibitory neurons in the cerebral cortex at P0 and in adult animals (Extended Data Fig. 7a–d,f–h), *Ebf1*^+^ projection inhibitory interneurons at P0 (Extended Data Fig. 7e) or the number of motor neurons in adult animals (Extended Data Fig. 7i–k), suggesting that the minor differences detected by snRNA-seq might be due to differences in the regional dissection. Thus, we were unable to detect a significant effect of ablating dOLCs on other cell types and, therefore, concluded that the locomotor and cognitive phenotype were most likely caused by changes in OLCs alone.

### eOLs in dorsal forebrain are distinct from original OLs

**Fig. 5:**
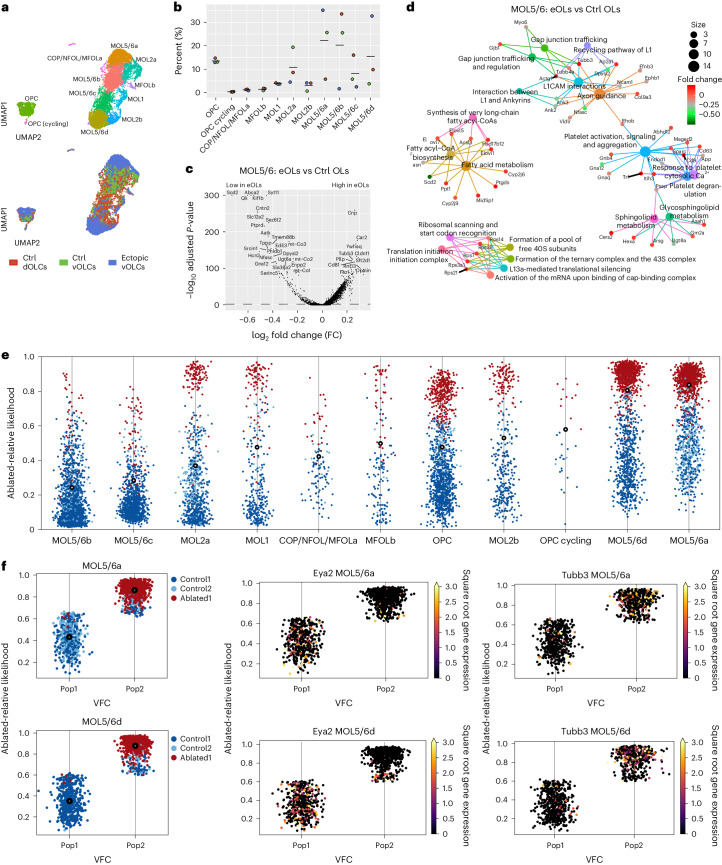
Ablation of dOPCs alters the transcriptional profiles of OL subpopulations. scRNA-seq of SOX10^+^ OLCs in the neocortex of adult control (*n* = 4) and ablated (*n* = 2) mice (P90). **a**, UMAP of all recovered cell clusters, sorted by cell population (top) or origin of cells (bottom) (*N* = 9,291 cells). COP, committed oligodendrocyte precursor; MFOL, myelin-forming oligodendrocyte; NFOL, newly formed oligodendrocyte. **b**, Frequency distribution analysis of cells in each cluster shown in **a** according to cell origin. **c**, Differential gene expression analysis of control versus ectopic OLs in cluster MOL5/6 shown in Extended Data Fig. 8a. Wilcoxon rank-sum test. **d**, Reactome pathway analysis of control versus ectopic OLs in cluster MOL5/6 shown in Extended Data Fig. 8a. **e**, Distribution of ablated-relative likelihood in each cluster shown in **a**. Dots in red and light red correspond to ablated-like cells, and blue and light blue indicate control-like cells. The gray circles represent the mean ablated-relative likelihood for each cluster. **f**, Ablated-relative likelihood highlighting ablation-unique transcriptional signatures within MOL5/6a (top) and MOL5/6d (bottom) populations. Left section of each panel represents cluster dissociation in two populations with unperturbed cells as Population1 and perturbated cells as Population2. Control-derived cells are labeled in blue and ablated cells in red. Right sections of each panel indicate expressions of gene markers for control-like population (Pop1) and ablated-like population (Pop2), as *Tubb3* and *Eya2*, respectively, with a maximum cutoff set at 3 for the square root transformed counts.

**Extended Data Fig. 8:**
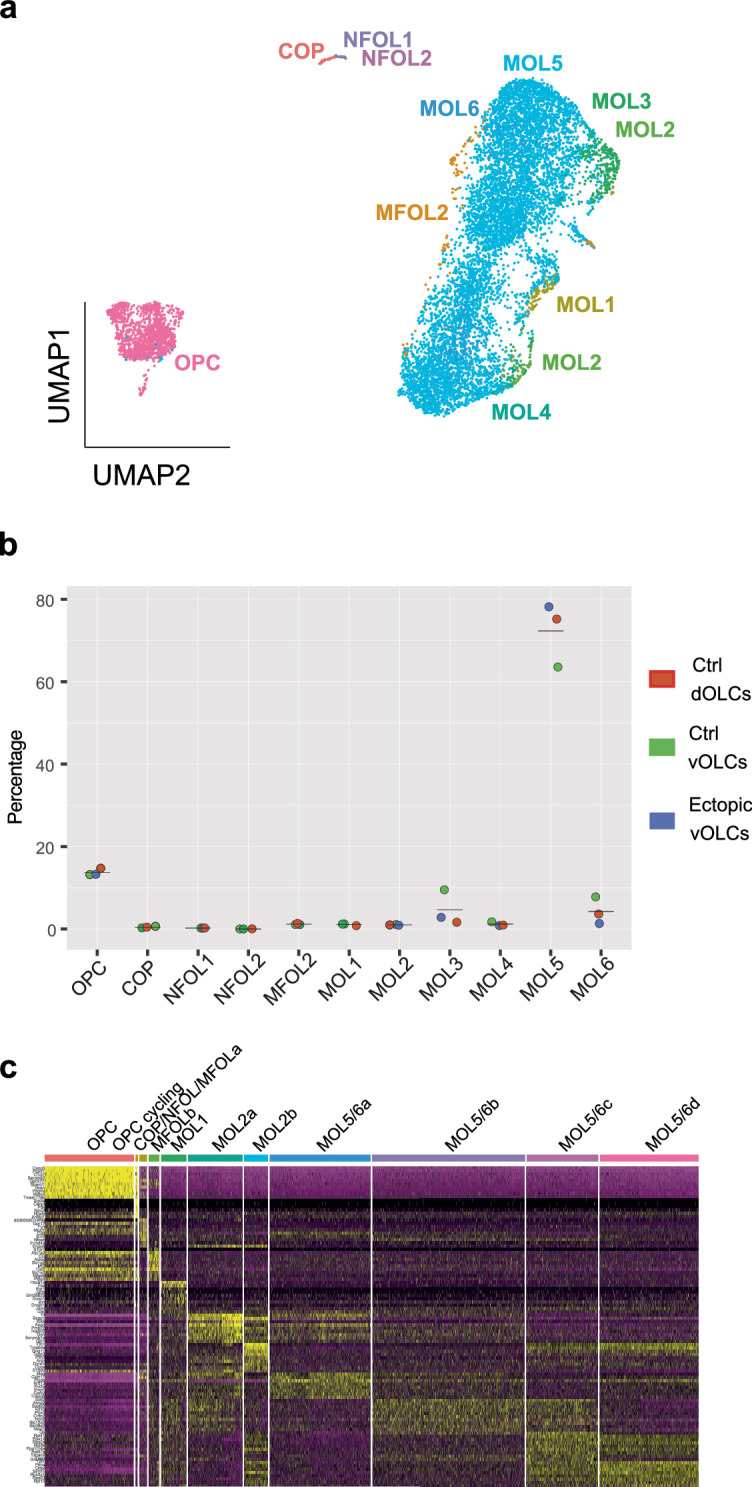
Global and population specific transcriptional responses to ablation. scRNA-sequencing of SOX10+ OLCs in the neocortex of adult mice (P90). (**a**) Uniform Manifold Approximation and Projection (UMAP) of all recovered cells clusters annotated according to 23. (**b**) Frequency dot plot of relative percentages of cluster sizes compared to all cells captured per sample. Control: n = 4, ablated: n = 2. (**c**) Heatmap illustrating the 10 top-most genes found per cluster. (Wilcoxon-rank sum-test of cluster against background).

**Extended Data Fig. 9:**
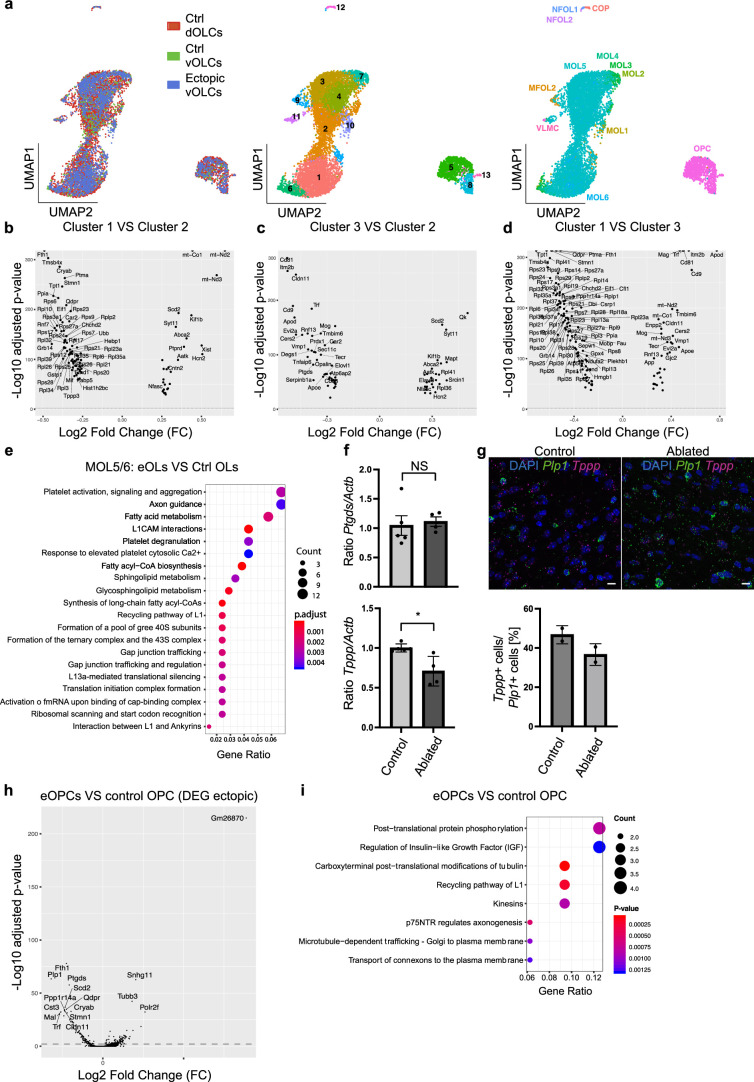
Integration of the scRNA-seq data using canonical correlation yielded similar gene expression differences between control and ectopic OLs. scRNA-sequencing data shown in Fig. 5 was integrated using Canonical Correlation Analysis (CCA). (**a**) Uniform Manifold Approximation and Projection (UMAP) of all recovered cell clusters, sorted by the origin of cells (left), clusters determined by Leiden clustering analysis (middle) or cell population label transfer classification (right). (b-d) Differential gene expression analysis of cluster 1 (enriched for ectopic OLs) versus cluster 2 (enriched for control OLs (**b**), cluster 3 (enriched for ectopic OLs) versus cluster 2 (enriched for control OLs) (**c**) and cluster 1 versus cluster 3 (both enriched for ectopic OLs) (**d**). Wilcoxon-rank sum-test. (**e**) Reactome pathway analysis of control versus ectopic OLs in cluster MOL5/6 shown in Extended Data Fig. 8a. Wilcoxon-rank sum-test. (**f**) qPCR analysis of Ptgds and Tppp gene expression between control and ablated animals. N = 3, control: n = 5, ablated: n = 4, control: p = 0.754, ablated: p = 0.011, unpaired t-test (two-sided). Data are represented as mean ± S.E.M. (**g**) RNA FISH analysis of Tppp mRNA expression between control and ablated animals. Control: N = 47, ablated: N = 95, n = 2. Scale bar: 10 µm (**h**) Differential gene expression analysis of control versus ectopic OPCs (see Fig. 5a). Wilcoxon-rank sum-test. (**i**) Reactome pathway analysis of control versus ectopic OPCs. Wilcoxon-rank sum-test.

To investigate whether the locomotor and cognitive deficits were due to a failure of the ectopic ventral oligodendrocyte lineage cells (vOLCs) to fully compensate for the dOLC populations that they replace, we performed single-cell RNA sequencing (scRNA-seq) of FACS-sorted SOX10^+^ OLCs from the adult neocortex (the predominant location of dOLCs and implicated in the behavioral tests) of control (*Emx/tdTom*) and ablated (*Emx/tdTom/DTA*) mice (Fig. 5 and Extended Data Figs. 8 and 9). dOPCs and vOPCs gave rise to all mature oligodendrocyte (MOL) subtypes with similar frequencies and mainly to MOL5 and MOL6 (MOL5/6) (Extended Data Fig. 8a–c), as previously identified^29,30^. However, cell clustering analysis revealed novel ablation-specific MOL5/6 subclusters (MOL5/6a and MOL5/6d) with different relative contributions based on their origin (Fig. 5a,b). Differential gene expression with Wilcoxon rank-sum test indicated that eOLs in MOL5/6 have a distinct transcriptional profile compared to control dOLs, with decreased expression of genes involved in the L1CAM pathway, fatty acid metabolism and sphingolipid metabolism, among other biological processes (Fig. 5c,d, Extended Data Fig. 9a–e and Supplementary Tables 1 and 2). We also performed perturbation analysis with MELD^28^ and confirmed that MOL5/6a and MOL5/6d are most perturbed by ablation of the dorsal OPCs (Fig. 5e), with genes *Tubb3* being upregulated and *Eya2* being downregulated (Fig. 5f). Among the genes downregulated in eOLs in the scRNA-seq data was *Tppp*, whose ablation in OLs phenocopies the myelin deficits observed after dorsal OPC ablation, namely reduced myelin sheath length and thickness, without affecting overall sheath number^31^. qPCR and RNA fluorescence in situ hybridization (FISH) validation confirmed that *Tppp* is downregulated in OLs isolated from control and ablated animals (Extended Data Fig. 9f,g). In addition, there was decreased expression of genes involved in the modulation of post-translational protein modifications, responsiveness to growth factors and transport of macromolecules in eOPCs compared to control dOPCs (Extended Data Fig. 9h,i and Supplementary Tables 1 and 2). Thus, eOLCs acquired transcriptional profiles distinct from the normal dOLCs that they replace, causing subtle myelination changes, which may explain the ablation-associated locomotor and cognitive changes.

## Discussion

Here we show that, although OLCs can populate and myelinate central nervous system regions that are normally the domain of OLCs of separate developmental origin, they cannot do so in a way that ensures optimal function. Specifically, myelination of the neocortex predominantly by OLCs of ventral origin rather than dorsal origin leads to deficits in aspects of locomotor and cognitive behavior. From this result, we conclude that the distinct developmental origins within the OL are essential for optimal central nervous system function.

The elimination of alternative explanations is critical to drawing this conclusion. First, could the phenotypes arise because of ablation-related changes in the tempo of myelination? The developing cortex is initially populated by OPCs of ventral origin before dorsal OPCs replace ventral OPCs after P0 (ref. ^13^). The initial population with ventral OPCs likely explains why, even by P3, most OLCs in the cortex of ablated animals are of ventral origin. Because there was no significant difference in OLC numbers and OPC proliferation postnatally between control and ablated animals, it is likely that ventral OPCs populate the cortex before birth and are not replaced when dorsal OPCs are ablated, confirming the previous observations by Kessaris et al.^13^. The overall numbers of OLCs and the onset of myelination were unchanged by dOLC ablation (Figs. 1 and 2), indicating that a delay in myelination is an unlikely explanation for the observed behavioral phenotypes. Second, could the functional changes that we observe arise due to non-cell-autonomous changes to non-oligodendrocyte lineage cells occurring because of the ablation process? We found no evidence of changes to either astrocytes or microglia in ablated animals, even comparing snRNA-seq data. However, we cannot fully exclude the possibility that inflammation after dOPC ablation that could influence neurodevelopmental processes occurs at an undetectable level or at a time that we have not captured^32^. We did, however, detect subtle changes in cortical neurons by snRNA-seq, and we cannot fully exclude that the observed changes in the number of projection inhibitory interneurons in ablated animals during development might contribute to the behavioral changes observed in ablated adult mice. In situ hybridization analysis of the cortex in ablated animals did not, however, support the indication of changes in this population of cells, where the ratio of excitatory versus inhibitory neurons and the number of *Ebf1*^+^ projection inhibitory neurons was unchanged in ablated animals. Taken together, these data suggest that the neuronal changes observed at P0 are unlikely to have contributed significantly to the locomotor and behavioral phenotype.

It is interesting to speculate why ventral OLCs cannot optimally support neuronal networks in the cortex. scRNA-seq analysis of the eOLCs showed a different transcription profile to OLCs normally populating the adult cortex (Fig. 5). Pathway analysis of the differentially expressed genes in OLs revealed dysregulation of pathways known to play a role in myelination^33–36^. For example, knockout of *Tppp*, a gene downregulated in eOLs, leads to impaired microtubule nucleation, resulting in aberrant myelination and motor coordination defects^31^. Thus, it is plausible that the reduced expression of *Tppp* in eOLs, together with the subtle dysregulations of a constellation of other genes important in myelination identified in this study, can explain the observed myelin phenotypes. Further detailed investigations are needed to determine if and how the dysregulated pathways can result in the observed behavioral changes. Although the overall level of myelination and the compaction of myelin appeared normal, we did observe slightly thinner myelin sheaths around small caliber axons and shorter paranodes in ablated animals. These subtle myelin changes may lead to altered neuronal circuit wiring, altered trophic support or changes in conduction velocity, resulting in the reported behavioral deficits. Relating the consequences of myelin changes to neuronal conduction, trophic support and circuit formation is technically very challenging, at least in part because of the difficulties inherent in pinpointing the cortical area relevant for the complex behavior changes that we observed in the ablated mice. Moreover, to date, no experimental tool is available to replicate the observed myelin sheath thinning, thus precluding us from investigating whether the subtle myelin changes indeed could cause the behavioral phenotypes.

OPCs have been implicated in the pathophysiology of some neurodevelopmental diseases, including schizophrenia^37^ and autism^38^. Thus, it is tempting to hypothesize about the clinical implications of the importance of the existence of the developmentally distinct OLC populations. Paramount to any clinical implications of the described findings is the existence of developmentally distinct OLC populations in humans. Multiple studies in developing human tissue point to the existence of vOLCs and dOLCs in humans^39–44^, albeit there is a lack of unequivocal proof due to the inability to label human cells over time. Independent genome-wide association studies showed that single-nucleotide polymorphisms (SNPs) in *Emx1*, the marker gene of dorsal origin, are associated with schizophrenia, whereas the marker gene of ventral origin lacks any disease association^45^. Therefore, it is possible that impaired dOPC development might contribute to schizophrenia.

In summary, we found that the ablation of dOLCs leads to the myelination of dorsal brain areas by vOLCs. These eOLCs show subtle differences in gene expression and myelin formation, most likely resulting in locomotor and cognitive behavioral changes in animals in which dOPCs were ablated. We, therefore, provide an explanation for OL developmental heterogeneity by revealing the critical relationship between spatiotemporal origin and function in the OL lineage.

## Methods

### Transgenic mouse lines

*Sox10-loxP-eGFP-polyA-STOP-loxP-tdTomato* (hereafter abbreviated as ‘*Sox10-GFP-tdTom*’ *or* ‘*Tom*’) reporter mice^15^ express enhanced GFP in all OLCs in the absence of Cre activity. To differentially label vOLCs and dOLCs in the forebrain, *Sox10-GFP-tdTom* mice were crossed to *Emx1-Cre* mice (hereafter abbreviated as ‘*Emx*’)^13^. To study the functional importance of dOLCs in the adult brain, dorsally derived, Emx1^+^ OLCs were ablated by crossing the *Emx1-Cre* line to the *Sox10-loxP-GFP-poly(A)-STOP-loxP-DTA* (hereafter abbreviated as ‘*Sox10-DTA*’ or ‘*DTA*’) mouse line^13^. To determine the ablation efficiency, all three mouse lines were crossed (hereafter abbreviated as ‘*Emx/Tom/DTA*’), allowing the visualization of the remaining dOLCs after ablation.

All mice were on a C57BL/6-B6CBA mixed background. Breeding colonies were maintained by mating transgenic mice with wild-type B6CBA-F1 mice. Mice were fed a standard diet and were kept on a 12-h dark/light cycle in individually ventilated cages. Mouse cages were kept at 45–65% humidity and at a temperature range of 20–24 °C. Experiments were performed on either neonatal (P0) or adult (11–14 weeks) animals. In case of the behavioral test, experiments were started at the age of 12 weeks. Mice ages are clearly stated in the text and respective figure legends. For cognitive behavior experiments, only male mice were used. For all other experiments, animals from both sexes were used. All animal experiments conformed to the UK Animals (Scientific Procedures) Act of 1986 and were approved by the Cambridge University local ethics committees before licensing by the UK Home Office.

### Behavioral tests

#### Open field test (general vigilance)

Individual mice were placed in the middle of an opaque, white plastic box (width, 50 cm; length, 50 cm), and their behavior was video-recorded for 5 min. The time spent close to the walls versus the center of the testing chamber was quantified.

#### Rearing test (general vigilance)

Individual mice were placed in an open-top, clear plastic cylinder (diameter, 15 cm; height, 30 cm), and their behavior was video-recorded for 1 min. The number of rears (both freely and against the wall) was counted.

#### Hanging bar test (forelimb strength)

For the hanging bar test, a metal bar (diameter, 6 mm) was elevated 30 cm above the table top. Individual mice were positioned on the bar in such a way that only the front paws grabbed onto the bar. If a mouse failed to grab the bar due to bad placement, the experiment was repeated after a brief rest. If a mouse fell within 5 s (not due to poor placement), the experiment was repeated up to three times. Mice were tested on three alternate days (one run per session). The time at which the mice fell off or touched the frame was scored. The following scoring system was used: Score 1 = 1–5 s, Score 2 = 6–10 s, Score 3 = 11–20 s, Score 4 = 21–30 s and Score 5 = >30 s.

#### Rotarod (locomotor coordination)

Individual mice were placed on a rotarod (Ugo Basile) facing forward. The rotarod was operated in acceleration mode, consistently accelerating from 4 r.p.m. to 40 r.p.m. over the course of 5 min. The speed at which a mouse fell off and the reason for the end of the trial (falling, jumping or passive rotation) were recorded.

#### Balancing beam (locomotor coordination)

For the balancing beam test, a metal beam (length, 1 m; diameter, 10 mm) was elevated 50 cm above the table top. Mice were trained to cross the beam independently once a day (2–3 test runs per session) for 1 week. Subsequently, mice were tested on three alternate days (two runs per session). All test runs were video-recorded. The time a mouse needed to cross the beam (time a mouse stood still on the beam was not considered) and the number of foot faults were counted.

#### Horizontal ladder (locomotor coordination)

For the horizontal ladder test, a ladder (length, 1 m; width, 10 cm) with evenly spaced rungs was elevated 50 cm above the table top. Mice were trained to cross the ladder independently once a day (2–3 test runs per session) for 2 weeks. On the test day, three runs of each mouse were video-recorded. The time a mouse needed to cross the ladder (time a mouse stood still on the ladder was not considered), the number of steps taken with one hindlimb and the number of slip-offs or misses of paw placement were counted.

#### Vertical beam (bradykinesia)

For the vertical beam test, a metal beam (length, 1 m; diameter, 20 mm) was embattled perpendicular to the table top. The vertical beam test does not require any training. The mice were tested on three alternate days (two runs per session). All test runs were video-recorded. The number of steps taken with the hindpaws were counted.

#### Gait analysis (gait)

The paws of mice were colored with ink to trace their gait on white paper. Mice were trained to walk continuously through a tunnel over white paper once a day (2–3 test runs per session) for 2 weeks. Subsequently, mice were tested on three alternate days (one run per session). Stride length and stride width were measured. Measurements were taken only if the animal walked at a constant speed for at least three body lengths (approximately 30 cm).

#### Von Frey test (sensation)

One hindpaw of individual mice was touched with continuous force using the electronic Von Frey apparatus (Ugo Basile) until the mouse pulled away the paw. The maximum force needed to provoke a reaction to the stimulus was recorded. From each animal, five measurements were taken.

#### rCPT (sustained attention)

Testing was performed in a touchscreen operant chamber for mice (Campden Instruments) as previously described^46^. After habituation, mice were trained to discriminate a visual ‘target’ stimulus from multiple ‘non-target’ stimuli in four stages as previously described^24^. After successfully completing the training stages, the performance of mice in the rCPT was assessed using the following basic parameters: stimulus set, one ‘target’ and four ‘non-target’ stimuli; SD time, 2 s; inter-trial interval, 5 s. Subsequently, to increase the load on attention, the SD (amount of time a stimulus was shown on the screen) was shortened to 1.5 s, 1.0 s and 0.5 s.

Four main measures of task performance were analyzed: response to the ‘target’ stimulus (hit), no response to ‘target’ stimulus (miss), response to ‘non-target’ stimulus (false alarm) and no response to ‘non-target stimulus (correct rejection). From these measures, the HR and FAR were calculated as follows:

$$\begin{array}{l}{\rm{HR}}=\frac{{\rm{Hit}}}{{\rm{Hit}}+{\rm{Miss}}}\qquad\qquad{\rm{FAR}}=\frac{{\rm{False}}\; {\rm{alarm}}}{{{\rm{False}}\; {\rm{alarm}}}+{{\rm{Correct}}\; {\rm{rejection}}}}\end{array}$$

Additionally, based on the HR and FAR, the sensitivity (d′), referring to the behavioral discrimination between ‘target’ and ‘non-target’ stimulus, and response bias (c), were calculated as follows:

$$\begin{array}{l}{\rm{d}}{\rm{\mbox{'}}}={\rm{z}}({\rm{HR}})-{\rm{z}}({\rm{FAR}})\qquad\qquad{\rm{c}}=-\frac{{\rm{z}}\left({\rm{HR}}\right)+{\rm{z}}({\rm{FAR}})}{2}\end{array}$$

c reflects the decision criterion used by the animal when deciding whether or not to respond. Thus, a low c indicates a bias of the animal to judge a stimulus as a target; a high c indicates a bias of the animal to judge a stimulus as a non-target.

#### Reversal learning

Reversal learning was carried out as previously described^47^. In brief, animals were trained to discriminate between a ‘target’ visual stimulus and a ‘non-target’ visual stimulus that were presented simultaneously side by side in two touchscreen windows. The correct response to the ‘target’ stimulus was always reinforced, whereas the response to the ‘non-target’ stimulus was never reinforced. Stimulus location (left or right) was pseudorandomly determined with the same spatial configuration never presented more than three times in a row. When all animals achieved a high level of performance (>80% correct choice for one session), these contingencies were reversed so that the previously correct ‘target’ stimulus became the incorrect ‘non-target’ stimulus and vice versa. Animals were tested daily on this reversal learning stage until performance reached higher than 80%. Key performance variables were the percentage of correct responses to the ‘target’ stimulus and the number of correction trials.

#### Extinction learning

Extinction learning was carried out as previously described^46^. In brief, all animals were initially trained to emit responses to a white stimulus located in the central window of a ‘3-choice’ Perspex mask. All responses were reinforced at this stage. Subsequently, extinction learning was assessed by identical task presentation but with omission of reinforcement. Data are expressed as the percentage of responses to a stimulus per session.

### scRNA-seq and snRNA-seq

#### Tissue dissociation and scRNA-seq

Neocortices of P90 *Emx/tdTom* (*n* = 4) and *Emx/Tom/DTA* (*n* = 2) were microdissected, and the tissue was dissociated into single-cell suspensions, as previously described^29^. In brief, mice were transcardially perfused with ice-cold oxygenated artificial cerebrospinal fluid (22 mM NaCl, 0.63 mM KCl, 0.4 mM NaH_2_PO_4_ × 2H_2_O, 6.5 mM NaHCO_3_, 25 mM saccharose, 5 mM glucose, 0.5 mM CaCl_2_, 4 mM MgSO_4_, pH 7.3), and the brains were collected. The tissue was sectioned at the vibratome in ice-cold artificial cerebrospinal fluid, and the CC was microdissected. Tissue dissociation was performed with an Adult Brain Dissociation Kit (Miltenyi Biotec) following the manufacturer’s instructions (the red blood cell removal step was not included). After debris removal, the cells were resuspended in ice-cold 1% BSA in artificial cerebrospinal fluid, filtered with a 30-μm filter (Sysmex Partec) and FACS sorted with a BD Influx system (BD Biosciences) to separate GFP^+^ and TdTom^+^ OLCs. An scRNA-seq experiment set with a cohort of control and experimental mice was discarded due to strong batch effects, most likely due to stress during the FACS procedure.

The sorted cells were processed with the Chromium Single Cell A Chip kit v2 and library prep with the Chromium Single Cell 3′ Library & Gel Beads kit v2 (10x Genomics) according to the manufacturer’s instructions. A total of 3,000 cells for each sample were loaded on the Chromium Single Cell A Chip, although a lower number of cells was recovered in singlet and passed the quality control.

#### Tissue collection and preparation for snRNA-seq

To assess the impact of dOPC ablation immediately when ablation occurs, cortical tissue was analyzed using snRNA-seq of antibody-hashed samples^26^. Neonatal mice (*Emx/tdTom* (*n* = 2) and *Emx/Tom/DTA* (*n* = 2)) were euthanized on the day of birth by decapitation, and then the motor and cingulate cortex was dissected in ice-cold oxygenated cutting solution containing 87 mM NaCl, 2.5 mM KCl, 1.25 mM NaH_2_PO_4_, 26 mM NaHCO_3_, 0.5 mM CaCl_2_, 4 mM MgSO_4_, 75 mM sucrose and 20 mM glucose, before samples were snap frozen on dry ice in a sterile microtube. The samples were stored at −80 °C until further processed. To isolate nuclei, tissue fragments from two animals with identical genotype were combined in nuclei isolation buffer (250 mM sucrose, 25 mM KCl, 5 mM MgCl_2_, 10 mM Tris buffer, pH 8.0, 0.1% NP40, 0.1 mM DTT and 0.4 U μl^−1^ RNAse inhibitors) and gently homogenized with a plastic pestle on ice. Subsequently, the homogenate was filtered using a 40-μm strainer, and the nuclear pellet was collected upon centrifugation. The pellet was then washed twice with Nuclei Wash and Resuspension (NWR) buffer containing 2% BSA in PBS and 0.4 U μl^−1^ RNAse inhibitors. Finally, the nuclei were resuspended in ST Staining Buffer (ST-SB: 2% BSA, 0.02% Tween 20, 10 mM Tris, pH 7.5, 146 mM NaCl, 1 mM CaCl_2_ and 21 mM MgCl_2_), filtered again with a 40-μm strainer and collected for inspection and counting. Hashtag antibody tagging was done as described previously^26^ with minor adjustments. Two sets of ablated and two sets of control nuclear samples (1 M nuclei per 100 μl of ST-SB) were first blocked with TruStain FcX (anti-mouse CD16/32, BioLegend, 101319) before the addition of 1 μg per 100 μl of Mab414 nuclei hashing antibodies (TotalSeq A0451-54, BioLegend, 682205, 682207, 682209 and 682211). To remove the unbound antibodies, samples were washed twice with ST Wash Buffer (10 mM Tris, 146 mM NaCl, 1 mM CaCl_2_, 21 mM MgCl_2_, 2% BSA, 0.4 U μl^−1^ of RNAse inhibitor) and again with NWR buffer to avoid the detergent and magnesium carry-over before sequencing. Finally, the nuclei were resuspended in NWR and strained with a 20-μm and a 10-μm strainer before counting, sample pooling and downstream processing with the 10x platform for single-nuclei sequencing.

### snRNA-seq bioinformatic analysis

#### Feature selection and antibody demultiplexing

Cell Ranger software (10x Genomics standard pipeline, version 3.1.0) was run twice on the single-nucleus dataset, first to unravel pre-mRNA expression of genes supported by mm10 genome and second to associate barcoded antibodies to cells, given as a list of features. Intersection of cell barcodes from both modalities was retained. Cells expressing more than 600 genes, with unique molecular identifiers (UMIs) higher than 1,000 and lower than 30,000, were selected. Moreover, cells with more than 1% of RNA counts being associated to mitochondrial environment were removed. After a centered log-ratio transformation, sample of origin assignation was performed for each cell by the HTODemux function from Seurat version 4.0, with a positive quantile value to consider a cell positive set at 0.99. Among the 35,820 valid cells processed by Cell Ranger, 14,294 were targeted by more than one unique antibody barcode; 10,474 cells were classified as negative, not reaching the positive quantile threshold; and 11,052 cells considered as barcoded by a unique antibody were saved for downstream analysis. A total of 5,198 cells were retained for the ablated condition (two antibodies labeling the ablated samples, 2,218 cells and 2,980 cells), and 5,854 cells were selected for the control condition (two antibodies labeling the control samples, 2,639 cells and 3,215 cells).

#### Clustering and label transfer

To cluster the remaining cells by features expression, dimensionality reduction was performed on log-transformed and scaled UMI counts of the 2,000 most variable features. After selection of the first 30 principal components by the elbow method, clustering was performed by a shared nearest neighbor graph based on *k*-nearest neighbors (KNN) calculated by Euclidean distances. Modularity optimization was done by the Louvain algorithm at a resolution of 0.6. The 19 clusters highlighted by this method were then analyzed and compared to previous single-cell transcriptomic datasets of developmental mouse brain^48,49^. Label transfer was performed with canonical correlation analysis using the Seurat functions FindTransferAnchors and TransferData. Final annotation of clusters was done in accordance with the outputted prediction scores from label transfers.

#### Markers

To unveil highly variable genes in each cell type, the Wilcoxon rank-sum test was used throughout FindAllMarkers function from Seurat, where significant genes (adjusted *P* value (*P*_adj_) < 0.01), expressed in at least 25% of cells in each population and with a log_2_ fold change higher than 0.25, were retained. Finally, the top 15 genes genes by cell type ranked by log_2_ fold change were selected to draw the heatmap.

#### Frequency analysis

For the sake of condition library size and cell type composition balance, the number of cells per condition and cell type was normalized to a percentage of each cell type per condition, divided by the number of conditions, allowing the assessment of cell representation across condition and cell type. We confirmed our findings by applying a Bayesian model for compositional single-cell data, scCODA version 0.1.8 (ref. ^27^), which highlighted no credible effects of the DTA perturbation on any of the cell population balance. Astrocyte population was selected as the reference cell type, which is assumed to be unchanged in absolute abundance, and parameters inference was calculated via Hamiltonian Monte Carlo (HMC) sampling.

#### Effect of experimental perturbation

To investigate whether each independent ablated cell presented hallmarks of transcriptional perturbation, we estimated the relative likelihood of observing each cell in each experimental condition. We computed the effect of experimental perturbation using MELD software version 1.0.0 (ref. ^28^). Following the guidelines presented in ref. ^26^, we performed L1 normalization on the raw counts for each cell, followed by a non-negative square root transformation. The potential of heat diffusion for affinity-based trajectory embedding (PHATE)^50^ was then drawn, with a KNN at 10 and an alpha decay parameter at 10, after principal component analysis (PCA) on the transformed counts. After benchmarking the optimal parameters to run the core MELD function, a beta at 67, a KNN at 7 and a mean square error at 0.001162 were used to generate the MELD graph from which estimation of densities of each sample were drawn. The densities were then normalized over sample to calculate the relative likelihood associated to each sample.

### scRNA-seq bioinformatic analysis

#### Feature selection

The count table from the output of the Cell Ranger software was obtained as part of the standard pipeline of the 10x Genomics platform (version 3.1.0). Cells expressing more than 200 genes but fewer than 3,000 were retained. Additionally, cells with more than 15% of RNA counts being associated mitochondrial origin were removed. Counts were normalized using the sctransform function in Seurat version 3.0 (refs. ^51,52^).

#### Clustering and label transfer

To cluster the cells, PCA was performed on the 3,000 most variable genes, and components were selected using the elbow method, selecting the first 30 principal components for further clustering. Cells were clustered using the Leiden algorithm in Seurat, with a resolution parameter of 0.6. To label the 11 clusters, we matched the labels to previous literature, where the backSPIN algorithm was used for clustering^29^. Label transfer was ultimately performed using the FindTransferAnchors and TransferData functions. With the clusters now labeled to their best matching cluster from the literature, our clusters were named accordingly. To integrate the data, the data were individually normalized per sample, and the FindTransferAnchors function was used to integrate the data.

#### Markers

The Wilcoxon rank-sum test was used to calculate the significant genes in relation to the obtained clusters. Significant genes (*P* < 0.01) were then selected. For each cluster, an enrichment score (z-score) was calculated using the FindAllMarkers function in Seurat, with a cutoff of 0.25 fold change.

#### Frequency analysis

The percentages of cells in the respective samples belonging to each cluster were calculated. The frequency graphs were then plotted as a percentage of the total captured cells in each sample, allowing us to make assessments on whether a certain sample is overrepresented or underrepresented compared to the other samples.

#### Differential gene expression analysis and Reactome pathway enrichment analysis

All comparisons were performed using the Wilcoxon rank-sum test with a Bonferroni-adjusted *P* value. Significantly differentially expressed genes with *P* < 0.01 and fold change > 0.1 were selected. Reactome pathways were calculated for each comparison using the R package ReactomePA^53^.

#### Effect of experimental perturbation

Similar to the snRNA-seq, we calculated the relative likelihood estimate of observing each cell in each experimental condition using MELD version 1.0.0. The same transformations were used on the raw counts to generate the PHATE projection, and the following parameters were drafted to generate the MELD graph (beta = 30, KNN = 8, mean square error = 0.004893). The densities were again normalized over sample to calculate the relative likelihood associated to each sample. For each cell type, we investigated the likelihood heterogeneity using vertex frequency clustering (VFC). We focused on two heterogeneous clusters, MOL5/6a and MOL5/6d, both of which were broken down in two populations according to their perturbated likelihood.

### Immunohistochemistry

Mice were terminally anesthetized and fixed by intracardiac perfusion using 4% (w/v) paraformaldehyde (PFA) in 0.01 M PBS. Tissue was post-fixed in 4% PFA for 2 h at 20–25 °C, cryoprotected with 20% (w/v) sucrose for 24–48 h, embedded and frozen in OCT medium and stored at −80 °C. Tissues were sectioned at 10 μm and collected onto poly-l-lysine-coated glass slides.

Tissue sections were incubated with blocking solution (10% (v/v) normal goat serum and 0.1% (v/v) Triton X-100 in PBS) for 1 h at 20–25 °C. In case antibodies raised in mouse were used, tissue sections were incubated with the MOM kit according to the manufacturer’s protocol (Vector Laboratories). In case the antigen required antigen retrieval for detection, slides were placed in antigen retrieval buffer solution (Dako, S2369, diluted 1:10 in distilled water), which was pre-heated to 95 °C, and slides were incubated for 10 min at 75 °C. Subsequently, tissue sections were incubated with primary antibodies overnight at 4 °C and then secondary antibodies for 1 h at 20–25 °C.

The following primary antibodies were used: CC-1 (anti-adenomatous polyposis coli (APC)) (OP80, mouse, 1:100, Calbiochem), CASPR1 (ab34151, rabbit, 1:1,000, Abcam), CD68 (MCA1957, rat, 1:200, Serotec), GFP (GFP-1020, chicken, 1:1,000, Aves Labs), GFAP (Z0334, rabbit, 1:1,000, Dako), IBA1 (019-19741, rabbit, 1:1,000, Wako), MBP (MCA4095, rat, 1:100, Serotec), NeuN (MAB377, mouse, 1:50, Millipore), tdTomato (AB8787-200, goat, 1:300, Sicgen Antibodies), OLIG2 (AB9610, rabbit, 1:1,000, Merck) and Ankyrin G (N106/36, mouse, 1:200, Antibodies Incorporated). The following secondary antibodies were used: anti-chicken Alexa 488 (donkey, 1:500, Jackson Laboratory), anti-goat Alexa 568 (donkey, 1:500, Thermo Fisher Scientific), anti-rabbit Alexa 647 (donkey, 1:500, Thermo Fisher Scientific), anti-rat Alexa 647 (donkey, 1:500, Thermo Fisher Scientific) and anti-mouse Alexa 647 (donkey, 1:500, Thermo Fisher Scientific). Cell nuclei were visualized by staining with Hoechst 33258 (1:10,000, Biotium). All images were acquired using an SP5 confocal microscope (Leica), except for the CASPR1 staining, which was imaged using an Airyscan microscope (Zeiss) to increase resolution. Cell counts were obtained from 3–6 biological replicates, 3–4 tissue sections per biological replicate. ImageJ (version 2.0.0-rc-68/1.52h and 1.53t), CellProfiler (version 2.2.0) and CellProfiler Analyst (version 2.2.1) were used for image analysis, cell counting and measurement of the size of the selected area.

### In situ hybridization

#### Assessment of OPC numbers

To visualize the distribution of OPCs, in situ hybridization with chromogenic RNAscope was performed. Tissue sections were incubated with RNAscope hydrogen peroxide reagent (ACDBio) for 10 min at 20–25 °C. Subsequently, tissue sections were permeabilized using RNAscope target retrieval reagent (ACDBio) for 5 min at 99–100 °C, followed by protease treatment using RNAscope Protease Plus (ACDBio) for 30 min at 40 °C. Probe hybridization, signal amplification and signal detection were performed using an RNAscope 2.5 HD Detection Kit (BROWN). The procedures were carried out according to the manufacturer’s instructions, except for the ‘AMP 5’ step, in which tissue sections were incubated for 45 min at 20–25 °C. The following probe was used: *Pdgfra* (mouse, 448431, ACDBio). Cell nuclei were visualized by staining with Hoechst 33258 (1:10,000, Biotium). Cell counts were obtained from five biological replicates, 3–4 tissue sections per biological replicate. ImageJ was used for image analysis, cell counting and measurement of the size of the selected area.

#### Assessment of neuronal subtype distribution and density at P90

Neuronal populations of the adult (P90) mouse cerebral cortex were characterized by single-molecule fluorescence in situ hybridization (smFISH) and immunohistochemistry for the neuron-specific marker RBFOX3 (NeuN). Three to five biological replicates were analyzed per genotype, with 2–4 whole-brain cryosections (coronal, 16 µm) per animal. As described previously^54^, smFISH staining was performed using an RNAscope LS Multiplex Fluorescent Assay (ACDBio; ref. ^55^), which was automated together with RBFOX3/NeuN immunohistochemistry on a BOND RX robotic stainer (Leica Biosystems), followed by spinning disk confocal imaging on an Operetta CLS high-content screening microscope (PerkinElmer) and image analysis using Harmony software (PerkinElmer).

RNAscope catalog probes (https://acdbio.com/catalog-probes) were assigned to four different channels (C1–C4; Supplementary Table 3) and combined in individual BOND runs for 3-plex or 4-plex transcript detection. RNAscope probes against bacterial *DapB* mRNA as well as against mouse housekeeping genes were used as negative and positive controls, respectively (Supplementary Table 3). The following modifications were applied to the previously published protocol^54^: for heat and protease treatment, P90 samples were incubated in BOND ER2 buffer, pH 9.0 (Leica Biosystems), at 95 °C for 5 min and in ACD protease reagent at 42 °C for 20 min. The probes in C1, C2 and C3 channels were labeled using Opal 520 (Akoya Biosciences (AB), FP1487001KT), Opal 570 (AB, FP1488001KT) and Opal 650 (AB, FP1496001KT) fluorophores, respectively, at a dilution of 1:1,500. The C4 probe complexes were developed with TSA biotin (AB, NEL700A001KT, 1:500) and streptavidin-conjugated Atto 425 (Sigma-Aldrich, 09260, 1:200). DAPI (Thermo Fisher Scientific, D1306, 0.25 μg ml^−1^) was used as nuclear counterstain. Slides were coverslipped using ProLong Gold Antifade Mountant (Thermo Fisher Scientific, P36930) and Precision cover glasses thickness No. 1.5H (Paul Marienfeld GmbH & Co. KG, 0107222).

For spinning disk confocal imaging, regions of interest (ROIs) for high-resolution ×40 scans, covering the cerebral cortex across individual hemispheres, were manually selected on low-magnification whole-tissue section ×5 scans. ×5 scans were acquired in wide-field mode with a numerical aperture (NA) 0.16 air objective; ×40 scans were acquired in confocal mode with a ×40 NA 1.1 automated water-dispensing objective. ×40 fields were imaged as a z-stack of 21 planes with a 1-μm step size and with 8% overlap.

To segment single neurons and quantify mRNA spots from high-resolution images, analysis scripts were created on Harmony software (PerkinElmer). Individual neurons were segmented as described previously^54^ using either *Synaptotagmin I* (*Syt1*) or RBFOX3/NeuN to delineate neuronal cytoplasm. The number of *Gad2* mRNA spots was calculated per *Syt1*^+^ single neuron for the identification of excitatory versus inhibitory neuron populations; the number of *Bcl11b* (*Ctip2*), *Crym* and *Fezf2* mRNA spots was calculated per NeuN^+^ single neuron for the identification of corticospinal motor neuron (CSMN) populations^56,57^. For neuronal subtype identification, the lowest point of the density curve of the respective spot count signal distribution, plotted on a logarithmic scale, was used for setting the cutoff: 15 mRNA spots for *Gad2*; five mRNA spots for *Bcl11b* (*Ctip2*), *Crym* and *Fezf2*. For example, a NeuN^+^ neuron with more than five *Fezf2* spots was classified as *Fezf2*^+^ and ≤5 *Fezf2* spots as *Fezf2*^−^.

Cortical layers and areas—that is, motor (MO) area and primary somatosensory (SSp) area—were manually annotated on coronal sections at the plane of the anterior corpus callosum. DAPI staining and layer neuron marker (*Cux2*, *Bcl11b*/*Ctip2* and *Foxp2*) expression patterns were used as anatomic landmarks and the Allen Reference Atlas (P56, coronal, image 44)^58^ as anatomic reference. Anatomic annotation masks were drawn on tissue maps, which were created by stitching ×40 maximum z-projection images in OMERO (University of Dundee & Open Microscopy Environment). Single-neuron objects were annotated with cortical layer and area identity based on their *x*–*y* position within anatomic annotation masks. For each cortical layer and area, the percentage (%) of each neuronal subtype across all *Syt1*^+^/NeuN^+^ neurons was calculated as well as neuronal subtype density (number of neurons per area (mm^2^)).

#### Assessment of neuronal subtype distribution at P0

For the assessment of neuronal subtypes in the P0 mouse cerebral cortex, the same protocol was followed as described above for P90, with the following exceptions: for heat and protease treatment, P0 samples were incubated in BOND ER2 buffer, pH 9.0, at 95 °C for 2 min and in ACD protease reagent at 42 °C for 10 min. Because of increased cell densities in the neonatal cerebral cortex, single cells were segmented from DAPI^+^ nuclear area plus a small cytoplasmic ring (1-µm perimeter around the DAPI^+^ nucleus), and RNA spot counts were evaluated in the segmented cell area. A cell with >5 *Syt1* spots in the segmented cell area was classified as a neuron; a neuron with >5 *Gad2* spots was classified as inhibitory and ≤5 *Gad2* spots as excitatory; and a neuron with >5 *Ebf1* spots was classified as *Ebf1*^+^.

#### RNA FISH

In situ four-color RNA FISH was performed using primer-padlock 2-oligo hybridization of the RNA targets, as described previously^59^, except that hybridization was performed in 30% formamide buffer (Merck). In brief, ssDNA oligo probes were designed against the coding regions of target RNA transcripts using Picky2.0 to identify loci without RNA secondary structure or repetitive sequence, followed by blast searching to confirm target specificity. Probe targets and sequences can be found in Supplementary Table 4. Probe hybridization was performed overnight at 40 °C with rocking in a humid chamber. Probes then underwent rolling circle amplification, polyacrylamide gel mounting and proteinase K (Sigma-Aldrich) sample clarification before imaging. Images were acquired on a Zeiss LSM 980 microscope.

#### Image analysis

Image analysis was performed in arivis Vision 4D, version 4.1.1. In the pipeline, nuclei were first segmented using DAPI staining. A 3-µm region grow was used to define the cell area. PLP^+^ cells were determined by mean intensity of GFP fluorescence per cell (threshold for positive cell: 400). Finally, individual *Tppp* dots larger than 0.5 µm were counted per cell. A cell with three or more *Tppp* dots was counted as positive.

### SCoRe and quantification

SCoRe imaging was performed to assess myelination status in motor cortex layer 1 as described previously^60,61^. In brief, mice were perfused transcardially under terminal anesthesia with 25 ml of warm (30 °C) PBS, followed by ice-cold 4% PFA. The brain was removed from the skull, and each hemisphere was dissected out and flattened by clamming between two glass slides with 1-mm spacer and continuing to fix in 4% PFA at 4 °C for 6 h before changing to PBS. The flat-mounted hemisphere was examined using a Zeiss LSM 880 laser scanning confocal microscope. Then, z-stack images (1,024 × 1,024 pixels) with a 2-mm z-step were captured from the outer surface of the motor cortex with a ×20 water immersion objective, and four tiled images were stitched into a larger view of the region. To quantify, three random ROIs of a volume 300 × 300 × 40 mm were selected and background subtracted, and the myelin signal was segmented and converted to a binary image with the Triangle algorithm in ImageJ. The total volume and length (skeleton) were calculated using Volocity software (Quorum Technologies). Internode length was measured semi-automatically on the image stacks using the ImageJ plugin Simple Neurite Tracer by manually locating adjacent nodes of Ranvier along continuous segments of myelin. A total of 294 internodes in six controls and 248 in five ablated animals were measured.

### Electron microscopy

Mice were perfused with 4% (w/v) glutaraldehyde in 0.01 M PBS. Tissue was post-fixed in 4% (w/v) glutaraldehyde at 4 °C overnight. The cortex was dissected, and the tissue was further processed in 2% osmium tetroxide at 4 °C overnight. The tissue was dehydrated in a series of ethanol washes (1× 70% EtOH for 15 min, 1× 95% EtOH for 15 min and 3× 100% EtOH for 10 min (Sigma-Aldrich)), washed twice in propylene oxide for 15 min and incubated in a 1:1 mix of propylene oxide and resin (50% resin, 34% dodecenyl succinic anhydride, 16% methyl nadic anhydride, 2% 2,4,6-Tris(dimethylaminomethyl)phenol (DMP-30), all (v/v), TAAB Laboratories) for at least 3 h at 20–25 °C. The tissue was incubated twice in pure resin for 12 h at 20–25 °C, and samples were embedded in plastic containers in fresh resin for 2 d at 60 °C. Ultra-thin sections of cortical layers 5–6 or CC were cut onto copper grids and stained with uranyl acetate and lead citrate (TAAB Laboratories). The sections were imaged with a H-600 Transmission Electron Microscope (Hitachi High-Tech). Subsequently, G-ratio (ratio of the inner axonal diameter to the total outer diameter) was measured from 100–500 axons per animal, 4–5 animals per age group and per genotype. The G-ratio measurement was carried out from images with magnification at ×3,000 or ×6,000, using the open-source software AxonSeg, with the built-in TEM model and artifact manually corrected^62^.

### Immunogold electron microscopy

Samples were prepared essentially as described previously^63^. In brief, mice were perfused after anesthesia transcardially with a fixing solution composed of 0.25% glutaraldehyde, 4% formaldehyde and 0.5% NaCl in phosphate buffer pH 7 according to Schultz and Karlsson^64^. After dissection and preparing horizontal vibratome sections of 300-µm thickness, the ROI (motor cortex layers 5–6) was cut out with a biopsy punch. These pieces of tissue were infiltrated overnight in 2.3 M sucrose in 0.1 M phosphate buffer pH 7, mounted on aluminum pins and frozen in liquid nitrogen. Ultra-thin cryosections were prepared using a diamond knife (cryoimmuno, 35°, Diatome) and a UC6 ultramicrotome equipped for cryosectioning (Leica Microsystems). Ultra-thin sections were placed on hexagonal 100 mesh copper grids (Science Services) and labeled for GFP (polyclonal rabbit, 1:100, Invitrogen, Thermo Fisher Scientific, RRID: AB_221569) or tdTom (polyclonal rabbit anti-RFP, 1:100, Rockland) followed by incubation with protein A-gold (10 nm, Cell Microscopy Core, UMC Utrecht). Images were taken with a LEO912 transmission electron microscope (Zeiss Microscopy GmbH) using an on-axis 2k CCD camera (TRS).

### Western blot

Whole-brain samples were lysed in IP lysis buffer (Thermo Fisher Scientific) supplemented with 0.1% Halt protease and phosphate inhibitor (Thermo Fisher Scientific). Tissue lysates were mixed with NuPAGE sample LDS loading buffer (Invitrogen) supplemented with 1× NuPAGE Reducing Agent (10x) (Invitrogen) and denatured for 10 min at 95 °C. Gel run and transfer were performed according to the manufacturer’s instruction for the mini gel tank (Thermo Fisher Scientific). After transfer, the nitrocellulose membranes were blocked in 1× Odyssey blocking solution (TBS, LI-COR) in TBS-T (TBS + 0.1% Tween (Sigma-Aldrich)) for 1 h at 20–25 °C on the shaker. Subsequently, nitrocellulose membranes were incubated with primary antibodies overnight at 4 °C and then secondary antibodies for 1 h at 20–25 °C. The following primary antibodies were used: β-ACTIN (A5441, mouse, 1:5,000, Sigma-Aldrich), GFAP (Z0334, rabbit, 1:1,000, Dako) and GS (ab73593, rabbit, 1:1,000, Abcam). The following secondary antibodies were used: anti-mouse 680 (donkey, 1:15,000, LI-COR) and anti-rabbit 800 (donkey, 1:15,000, LI-COR). Fluorescent signals were detected using the Odyssey apparatus (LI-COR) with an exposure time of 2 min. Band intensities of the developed western blots were determined with Image Studio software (version 4.0, LI-COR).

### Isolation of adult oligodendrocytes

Adult male and female mice (2–3 months) were decapitated after lethal injection with phenobarbital. The brains were removed quickly and placed into ice-cold Hibernate A (BrainBits). The meninges and the olfactory bulb were mechanically removed in Hibernate A, and the brain tissue was mechanically minced into 1-mm^3^ pieces. The tissue pieces were spun down at 100*g* for 1 min at room temperature, and the tissue was washed in HBSS^−^ (no Mg^2+^ and no Ca^2+^, Gibco). Each brain was mixed with 10 ml of dissociation solution (34 U ml^−1^ papain (Worthington) and 20 mg ml^−1^DNase Type IV (Gibco) in Hibernate A). The brain tissue was dissociated on a shaker (50 r.p.m.) for 40 min at 35 °C. The digestion was stopped by addition of ice-cold HBSS^−^. The tissue was centrifuged (200*g*, 3 min, room temperature); the supernatant was completely aspirated; and the tissue was resuspended in Hibernate A supplemented with 2% B27 (Gibco) and 2 mM sodium pyruvate (Gibco) (trituration solution). The tissue was allowed to sit in this solution for 5 min. To obtain a single-cell suspension, the tissue suspension was triturated 10 times using first a 5-ml serological pipette and subsequently three fire-polished glass pipettes (opening diameter >0.5 mm). After each trituration step, the tissue suspension was allowed to sediment (approximately 1–2 min), and the supernatant (approximately 2 ml), containing the cells, was transferred into a fresh tube. After each round of trituration, 2 ml of fresh trituration solution was added. To remove accidentally transferred undigested tissue bits, the collected supernatant was filtered through 70-μm cell strainers into tubes that contained 90% isotonic Percoll (GE Healthcare, 17-0891-01, in 10× PBS, pH 7.2 (Life Technologies)). The final volume was topped up with phenol red free DMEM/F12 with HEPES (Gibco) and mixed to yield a homogeneous suspension with a final Percoll concentration of 22.5%. The single-cell suspension was separated from remaining debris particles by gradient density centrifugation (800*g*, 20 min, room temperature, without break). The myelin debris and all layers without cells were discarded, and the brain cell containing phase (last 2 ml) and the cell pellet were resuspended in HBSS^+^ and centrifuged (300*g*, 5 min, room temperature). The cell pellet was resuspended in red blood cell lysis buffer (Thermo Fisher Scientific, 15838518) and incubated for 90 s at room temperature to remove red blood cells. Next, 10 ml of HBSS^+^ was added to this cell suspension and spun down (300*g*, 5 min, room temperature). The cell pellets were resuspended in 0.5 ml of modified Miltenyi washing buffer (MWB, 2 mM EDTA (Thermo Fisher Scientific), 2 mM sodium pyruvate, 0.5% BSA in PBS, pH 7.3) supplemented with 10 ng ml^−1^ human recombinant insulin (Gibco). To this cell suspension, 2.4 μg of Mouse MOG Biotinylated antibody (BAF2439, BioTechne) in 100 μl was added for every 10 million cells. After 25-min incubation, gently shaking at 4 °C, 7 ml of MWB was added. The solution was centrifuged (300*g*, 5 min, room temperature), and the pellet was resuspended in 80 μl of MWB supplemented with 20 μl of Anti-Biotin MicroBeads (Miltenyi Biotec, 130-090-485) per 10 million cells. The cells were incubated for 15 min, slowly shaking at 4 °C. The secondary antibody was again washed out with 7 ml of MWB, and the sample was centrifuged (300*g*, 5 min, room temperature). The cell pellet was resuspended in 0.5 ml, and MACS was performed according to the recommendations of the supplier. In brief, an MS column (Miltenyi Biotec, 130-042-201) was inserted into a MiniMACS Separator (Miltenyi Biotec, 130-042-102) and pre-wet with 0.5 ml of MWB. Resuspended cells were put onto one MS column. Subsequently, the column was washed three times using 500 ml of MWB for each wash. Finally, MOG^+^ cells were flushed out the column with 0.5 ml of MWB. Cells were centrifuged (300*g*, 5 min, room temperature), and then the pellet was resuspended in 500 μl of TRIzol (Thermo Fisher Scientific, 15596026) and frozen at −80 °C until RNA isolation.

### RNA isolation and RT–qPCR

RNA was isolated from freshly purified OLs according to the Direct-zol RNA MicroPrep Kit (Zymo Research, R2061). cDNA was generated using the Maxima First Strand Kit according to the manufacturerʼs instructions (Thermo Fisher Scientific; K1641). For RT–qPCR, the following primers were used: *Pdgfra*: FWD 5′-GCCTCCATTCTGGAGCTTGT-3′, REV 5′-CTCGATGTTCGGAAGAGGG-3′; *Mag*: FWD 5′-GGATAATGATTTCAGCTTCTCG-3′, REV 5′-GTAGGGACTATTGAAATACCAG-3′; *Tppp*: FWD 5′-ACGTGGACATTGTCTTCAGCA-3′, REV 5′-GAGGACACAGCTTTCGTGA-3′. All primers were used at a concentration of 400 mM. The efficiency of each primer was greater than 95% as determined for each primer pair by serial dilutions of OL cDNA. cDNA, primers and the Syber Green Master Mix (Thermo Fisher Scientific, A25741) were mixed as instructed by the manufacturer, and RT–qPCR and melting curve analysis were performed on a QuantStudio 7 Flex Real-Time PCR System (Life Technologies). Fold changes in gene expression were calculated using the Pfaffl method in Microsoft Excel.

### Statistics

In all, the graphical data are presented as mean ± s.e.m. Statistical analysis was performed using GraphPad Prism (versions 9 and 10). The data were analyzed for normal distribution using D’Agostino–Pearson omnibus and Shapiro–Wilk normality test. To assess differences between two groups, the two-tailed unpaired Studentʼs *t*-test or the Mann–Whitney non-parametric test was used, depending on whether the data were distributed normally or not. When comparing more than two groups, a one-way ANOVA test was used followed by Tukey’s post hoc test. If the sample was not normally distributed, a Kruskal–Wallis test combined with Dunn’s post hoc test was used. In case a dataset had two or more variables, a two-way ANOVA was performed. If an interaction of the variables in the dataset was detected in the two-way ANOVA, the variables were analyzed separately by one-way ANOVA combined with Tukey’s post hoc test. If the sample was not normally distributed, a Kruskal–Wallis test (ranks) was carried out. To analyze data obtained by repeated measurements (for example, rCPT, reversal learning and extinction learning), the mixed-effect model was performed using the function ‘lmer’ from the R package ‘lme4’. If an interaction of the variables in the dataset was detected in the mixed-effect model, each group was analyzed separately by simple main effect model.

For behavioral studies, pilot experiments were run on which basis the sample size was determined by power analysis. For all other experiments, no statistical methods were used for sample size determination. All experiments were performed with three or more replicates, unless stated otherwise, which are similar sample sizes to those standard in the field. For cell quantifications, a sufficient number of cells per animal was counted (≥50) to ensure normal distribution around the mean. No animals or data points were excluded from the analyses reported in this paper. There was no randomization when animal and samples were assigned to the various experimental groups, because groups were determined by an animal’s genotype. Experimental conditions were the same for each group.

Data collection was not performed blinded apart from the behavioral experiments, where the animal identity was coded before testing. Data analysis was performed blinded to the conditions of the experiments and automated wherever possible to limit the influence of the experimenter on the outcome. In each figure legend, the number of technical replicates (*N*), the number of biological replicates (number of animals) (*n*), the statistical test and the significance levels are indicated. Datasets were considered significantly different at a *P* value of less than 0.05. Significance levels are presented graphically as follows: *P < 0.05, **P < 0.01, ***P < 0.001 and ****P < 0.0001.

### Reporting summary

Further information on research design is available in the Nature Portfolio Reporting Summary linked to this article.

## Online content

Any methods, additional references, Nature Portfolio reporting summaries, source data, extended data, supplementary information, acknowledgements, peer review information; details of author contributions and competing interests; and statements of data and code availability are available at 10.1038/s41593-024-01666-8.

### Supplementary information

Reporting Summary

Supplementary Table 1 Differential gene expression analysis using the Wilcoxon rank-sum test.

Supplementary Table 2 Reactome pathway analysis.

Supplementary Table 3 List of RNAscope probes.

Supplementary Table 4 List of RNA FISH probes.

Supplementary Video 1 Horizontal beam test.

## Data Availability

RNA sequencing data that support the findings of this study are deposited in the Gene Expression Omnibus (GSE254447).

## Code availability

Code for single-nucleus and single-cell RNA sequencing analysis is available at https://github.com/Castelo-Branco-lab/DTablation2023.
