# Supplementary material for: Developmental origin of oligodendrocytes determines their function in the adult brain
Source: Nat Neurosci. 2024 Jun 7;27(8):1545–54. doi: 10.1038/s41593-024-01666-8 (PMC11303253; doi:10.1038/s41593-024-01666-8)
Supplement: Supplementary file 1 — Reporting Summary [file 41593_2024_1666_MOESM1_ESM.pdf]

nature research

Corresponding author(s): Prof Robin JM Franklin

Last updated by author(s): 23/02/24

# Reporting Summary

Nature Research wishes to improve the reproducibility of the work that we publish. This form provides structure for consistency and transparency in reporting. For further information on Nature Research policies, see Authors & Referees and the Editorial Policy Checklist.

## Statistics

For all statistical analyses, confirm that the following items are present in the figure legend, table legend, main text, or Methods section.

| n/a | Confirmed | |
|---|---|---|
| ☐ | ☒ | The exact sample size (*n*) for each experimental group/condition, given as a discrete number and unit of measurement |
| ☐ | ☒ | A statement on whether measurements were taken from distinct samples or whether the same sample was measured repeatedly |
| ☐ | ☒ | The statistical test(s) used AND whether they are one- or two-sided<br>*Only common tests should be described solely by name; describe more complex techniques in the Methods section.* |
| ☐ | ☒ | A description of all covariates tested |
| ☐ | ☒ | A description of any assumptions or corrections, such as tests of normality and adjustment for multiple comparisons |
| ☐ | ☒ | A full description of the statistical parameters including central tendency (e.g. means) or other basic estimates (e.g. regression coefficient) AND variation (e.g. standard deviation) or associated estimates of uncertainty (e.g. confidence intervals) |
| ☐ | ☒ | For null hypothesis testing, the test statistic (e.g. *F*, *t*, *r*) with confidence intervals, effect sizes, degrees of freedom and *P* value noted<br>*Give P values as exact values whenever suitable.* |
| ☐ | ☒ | For Bayesian analysis, information on the choice of priors and Markov chain Monte Carlo settings |
| ☒ | ☐ | For hierarchical and complex designs, identification of the appropriate level for tests and full reporting of outcomes |
| ☒ | ☐ | Estimates of effect sizes (e.g. Cohen's *d*, Pearson's *r*), indicating how they were calculated |

*Our web collection on statistics for biologists contains articles on many of the points above.*

## Software and code

Policy information about availability of computer code

| | |
|---|---|
| Data collection | No code has been used for data collection. |
| Data analysis | Statistical analysis: Graphpad Prism (Version 9 and 10)<br><br>Western Blot analysis: Image studio software (Version 4.0, Licor)<br><br>Image analysis (EM, IHC, ISH, RNA FISH): ImageJ (Version 2.0.0-rc-68/1.52h and 1.53t), Cell Profiler (Version 2.2.0), Cell profiler analyst (Version 2.2.1) and Arivis Vision 4D (version 4.1.1).<br><br>Single-nucleus RNA seq analysis: Feature selection and antibody demultiplexing: Cellranger software (version3.1.0), NormalizeData function (LogNormalize) (Seurat, v4.0.0), HTODemux (Seurat, v4.0.0) and NormalizeData function (CLR) (Seurat, v4.0.0); Clustering: Louvain algorithm, FindTransferAnchors and TransferData (Seurat v4.0.0); Marker analysis: FindAllMarkers (Seurat v4.0.0); Frequency analysis: scCODA, v0.1.8; Effect of experimental perturbation: MELD, v1.0.0<br><br>Single-cell RNA seq analysis: Feature selection: Cellranger software (version 2.1.1) and sctransform function (Seurat, v3.0.0); Clustering: Leiden algorithm, FindTransferAnchors and TransferData (Seurat v3.0.0); Marker analysis: FindAllMarkers (Seurat v3.0.0); Reactome pathway analysis: R-package ReactomePA, v1.30; Effect of experimental perturbation: MELD, v1.0.0<br><br>Code for single-nucleus and single-cell RNA seq analysis will be made available at https://github.com/Castelo-Branco-lab/DTablation2020. |

For manuscripts utilizing custom algorithms or software that are central to the research but not yet described in published literature, software must be made available to editors/reviewers. We strongly encourage code deposition in a community repository (e.g. GitHub). See the Nature Research guidelines for submitting code & software for further information.

## Data

Policy information about availability of data

All manuscripts must include a data availability statement. This statement should provide the following information, where applicable:

- Accession codes, unique identifiers, or web links for publicly available datasets
- A list of figures that have associated raw data
- A description of any restrictions on data availability

Data availability: RNA sequencing data that support the findings of this study are currently deposited on GEO (GSE254447).

# Field-specific reporting

Please select the one below that is the best fit for your research. If you are not sure, read the appropriate sections before making your selection.

☒ Life sciences ☐ Behavioural & social sciences ☐ Ecological, evolutionary & environmental sciences

For a reference copy of the document with all sections, see nature.com/documents/nr-reporting-summary-flat.pdf

# Life sciences study design

All studies must disclose on these points even when the disclosure is negative.

| | |
|---|---|
| Sample size | For behavioral studies pilot experiments were run, on which basis the sample size was determined by power analysis. For all other experiments no statistical methods were used for sample size determination. All experiments have been performed with three or more replicates, unless stated otherwise, similar sample sizes to those standard in the field. For cell quantifications, a sufficient number of cells per animal was counted (≥50) to ensure normal distribution around the mean. |
| Data exclusions | No data were excluded from the analysis. |
| Replication | All behavioral tests were repeated twice with independent cohorts of mice. All results were successfully replicated.<br>All other experiments were repeated in different biological replicates at least three times. All results were successfully replicated. |
| Randomization | There was no randomization when animal/samples were assigned to the various experimental groups, because groups were determined by animal's genotype. Experimental conditions were the same for each group. |
| Blinding | Data collection was not performed blind apart from the behavioral experiments, where the animal identity was coded previous to testing. Data analysis was performed blind to the conditions of the experiments and automated wherever possible limit the influence of the experimenter on outcome. |

# Reporting for specific materials, systems and methods

We require information from authors about some types of materials, experimental systems and methods used in many studies. Here, indicate whether each material, system or method listed is relevant to your study. If you are not sure if a list item applies to your research, read the appropriate section before selecting a response.

### Materials & experimental systems

| n/a | Involved in the study | |
|---|---|---|
| ☐ | ☒ | Antibodies |
| ☒ | ☐ | Eukaryotic cell lines |
| ☒ | ☐ | Palaeontology |
| ☐ | ☒ | Animals and other organisms |
| ☒ | ☐ | Human research participants |
| ☒ | ☐ | Clinical data |

### Methods

| n/a | Involved in the study | |
|---|---|---|
| ☒ | ☐ | ChIP-seq |
| ☒ | ☐ | Flow cytometry |
| ☒ | ☐ | MRI-based neuroimaging |

## Antibodies

| | |
|---|---|
| Antibodies used | Immunohistochemistry (IHC)<br>Ankyrin G: N106/36, mouse, 1:200, Antibodies Incorporated<br>CC-1: OP80, mouse, 1:100, Calbiochem<br>Caspr1: ab34151, rabbit, 1:1000, Abcam<br>CD68: MCA1957, rat, 1:200, Serotec<br>GFP: GFP-1020, chicken, 1:1000, Aves lab<br>GFP: A-11122, rabbit, 1:100, Invitrogen/ThermoFischer<br>GFAP: Z0334, rabbit, 1:1000, DAKO |

IBA1: 019-19741, rabbit, 1:1000, Wako
MBP: MCA4095, rat, 1:100, Serotec
NeuN: MAB377, clone A60, mouse, 1:50, Millipore
Olig2: AB9610, rabbit, 1:1000, Merck
tdTomato AB8787-200, goat, 1:300, Sicgen antibodies
tdTomato: 600-401-379, rabbit, 1:100, Rockland
anti-chicken Alexa 488: donkey, 1:500, Jackson Laboratory
anti-goat Alexa 568: donkey, 1:500, Thermo Fisher
anti-rabbit Alexa 647: donkey, 1:500, Thermo Fisher
anti-rat Alexa 647: donkey, 1:500, Thermo Fisher
anti-mouse Alexa 647: donkey, 1:500, Thermo Fisher

Western Blot (WB)
Actin: A5441, mouse, 1:5000, Sigma-Aldrich
GFAP: Z0334, rabbit, 1:1000, DAKO
GS: ab73593, rabbit, 1:1000, abcam
anti-mouse 680, donkey, 1:15000, Licor
anti-rabbit 800, donkey, 1:15000, Licor

Validation

Actin (Sigma-Aldrich): Validated for WB by Sigma-Aldrich. Cited in >5000 publications.
Ankyrin G (Antibodies Incorporated): Validated for IHC by Antibodies Incorporated. Cited in >200 publications.
CC-1 (Calbiochem): Widely used for IHC as shown for example in Huang et al., 2011, Nature Neuroscience, doi:10.1038/nn.2702
Caspr1 (Abcam): KO-validated by Abcam. Validated for IHCs. Widely used in > 40 references
CD68 (Serotec): Validate for IHC by Biorad. Cited in >40 publications.
GFP (Aves lab): Validated for IHCs by Aves (tested in transgenic mice expressing GFP). Cited in >40 publications.
GFP (Thermofisher): Validated for IHCs by Thermofisher. This antibody was verified by relative expression to ensure that the antibody binds to the antigen stated by Thermofisher. Cited in >1500 publications.
GFAP (DAKO): Validated for IHCs and WB by Dako. Cited in >300 publications.
GS (Abcam): Validated in WB and tested in mouse by Abcam. Cited in 25 publications. Independently reviewed in 11 reviews.
IBA1 (Wako): Validated in IHC by Sigma-Aldrich. Cited in 19 publications.
MBP (Serotec): Widely used as shown for example in Huang et al., 2011, Nature Neuroscience, doi:10.1038/nn.2702
NeuN (Millipore): Validated for IHC by Sigma-Aldrich. Cited in >100 publications.
Olig2 (Merck): Validated for IHC by Merck-Millipore. Cited in >100 publications.
tdTomato (Sicgen): Validated for IHC by Sicgen (tested in HEK cells transfected with TdTomato). Cited in 30 publications.
tdTomato (Rockland): Validated for IHC by Rockland. Validated for EM by scientific publications (e.g. PMID: 33833289). Cited in >900 publications.

## Animals and other organisms

Policy information about studies involving animals; ARRIVE guidelines recommended for reporting animal research

Laboratory animals

All mice are on a C57BL/6-B6CBA mixed background. Breeding colonies were maintained by mating transgenic mice with wildtype B6CBA-F1 mice. Mice were fed a standard diet and were kept on a 12 hr dark/light cycle in individually ventilated cages. Mouse cages was kept at 45 to 65% and a temperature range of 20-24°C. Experiments were performed on either neonatal (P0) or adult animals (11-14 weeks). In case of the behavioral test, experiments were started at the age of 12 weeks. Mice ages are clearly stated in the text and respective figure legends. For cognitive behaviour experiments only male mice were used.

The following mouse strains have been used in this study:
Sox10-loxP-eGFP-polyA-STOP-loxP-tdTomato: Tripathi et al., J Neurosci, 2011, PMID: 21543611
Emx1-Cre: Kessaris et al., Nat Neurosci, 2006, PMID: 16388308
Sox10-loxP-GFP-poly(A)-STOP-loxP-DTA: Kessaris et al., Nat Neurosci, 2006, PMID: 16388308

Wild animals

The study did not involve wild animals.

Field-collected samples

The study did not involve samples collected from the field.

Ethics oversight

All animal experiments conformed to the UK Animals (Scientific Procedures) Act 1986 and were approved by the Cambridge University local ethical committees before licensing by the UK Home Office (Project license: PC0C0F291).

Note that full information on the approval of the study protocol must also be provided in the manuscript.
